# Distinct Roles of Meiosis-Specific Cohesin Complexes in Mammalian Spermatogenesis

**DOI:** 10.1371/journal.pgen.1006389

**Published:** 2016-10-28

**Authors:** Uddipta Biswas, Kai Hempel, Elena Llano, Alberto Pendas, Rolf Jessberger

**Affiliations:** 1 Institute of Physiological Chemistry, Medical Faculty Carl Gustav Carus, Technische Universität Dresden, Dresden, Germany; 2 Centro de Investigacion del Cancer (CSIC-USAL), Campus Miguel de Unamuno, Salamanca, Spain; Cornell University, UNITED STATES

## Abstract

Mammalian meiocytes feature four meiosis-specific cohesin proteins in addition to ubiquitous ones, but the roles of the individual cohesin complexes are incompletely understood. To decipher the functions of the two meiosis-specific kleisins, REC8 or RAD21L, together with the only meiosis-specific SMC protein SMC1β, we generated *Smc1*β^*-/-*^*Rec8*^*-/-*^ and *Smc1β*^*-/-*^*Rad21L*^*-/-*^ mouse mutants. Analysis of spermatocyte chromosomes revealed that besides SMC1β complexes, SMC1α/RAD21 and to a small extent SMC1α/REC8 contribute to chromosome axis length. Removal of SMC1β and RAD21L almost completely abolishes all chromosome axes. The sex chromosomes do not pair in single or double mutants, and autosomal synapsis is impaired in all mutants. Super resolution microscopy revealed synapsis-associated SYCP1 aberrantly deposited between sister chromatids and on single chromatids in *Smc1β*^*-/-*^*Rad21L*^*-/-*^ cells. All mutants show telomere length reduction and structural disruptions, while wild-type telomeres feature a circular TRF2 structure reminiscent of t-loops. There is no loss of centromeric cohesion in both double mutants at leptonema/early zygonema, indicating that, at least in the mutant backgrounds, an SMC1α/RAD21 complex provides centromeric cohesion at this early stage. Thus, in early prophase I the most prominent roles of the meiosis-specific cohesins are in axis-related features such as axis length, synapsis and telomere integrity rather than centromeric cohesion.

## Author Summary

Unlike somatic cells, which feature two different cohesin complexes, in spermatocytes at least six distinct cohesin complexes form, whose concerted functions are little understood. This study focuses on three meiosis-specific cohesins. Meiosis features specific chromosome structures and dynamics, and we revealed individual contributions of meiotic cohesin complexes to chromosome axes length, centromeric cohesion, telomere integrity and synapsis. The only meiosis-specific SMC protein, SMC1β, was removed leaving only complexes based on the universal SMC1α. In addition to SMC1β, either one of the two meiosis-specific kleisins REC8 or RAD21L, proteins that close the cohesin ring-like structure, were eliminated. “Double-knockout” mutants were compared to the single “knockouts” and wild-type. Telomeres and chromosome synapsis are impaired to different degrees in all mutants. In early prophase I prominent roles of meiosis-specific cohesins are in axis length and synapsis rather than centromeric cohesion. Removal of SMC1β and RAD21L almost completely abolishes all chromosome axes. Centromeric cohesion is initially provided by SMC1α complex(es). Later in meiosis, SMC1β ensures centromeric cohesion, suggesting functional replacement of SMC1α. Thus, different cohesin complexes in spermatocytes contribute distinctly to different structures and processes in these cells, but there is also some functional redundancy.

## Introduction

After completing premeiotic DNA replication mammalian germ cells enter meiosis and undergo two meiotic cell divisions without any further DNA replication. Haploid gametes are produced. Meiosis features highly specific chromosome structures and behaviour to ensure proper chromosome segregation, exchange of genetic information, and maintenance of genome integrity (reviewed in [1]). In leptonema the four sister chromatids become increasingly compacted and each pair of sister chromatids forms an axial element (AE), most often characterized by the axial element proteins SYCP2 and SYCP3. The compacted AEs start to pair in zygonema, i.e. the two homologous pairs (homologs) of sister chromatids synapse and form the synaptonemal complex (SC), which is complete in pachynema. The SC thus contains four sister chromatids. Each pair of sister chromatids is held together by cohesins, the two pairs are embedded in SC proteins. Once synapsed, the AEs are called lateral elements (LEs) of the SC. The protein SYCP1 is centrally located in the SC between the LEs and serves as a marker for synapsis. Homologous recombination between the two homologs requires the introduction of programmed double strand breaks (DSBs) by the topoisomerase-type enzyme SPO11. These breaks, which can be visualized by staining for double-strand break repair proteins such as the meiosis-specific DMC1, are introduced in leptonema and are processed into recombination intermediates until pachynema. In diplonema the SC between homologs disassembles, the homologs desynapse, but remain linked through a few chiasmata, the sites of meiotic recombination, until the homologs are separated in anaphase of meiosis I and the recombination process is completed.

**Fig 1:**
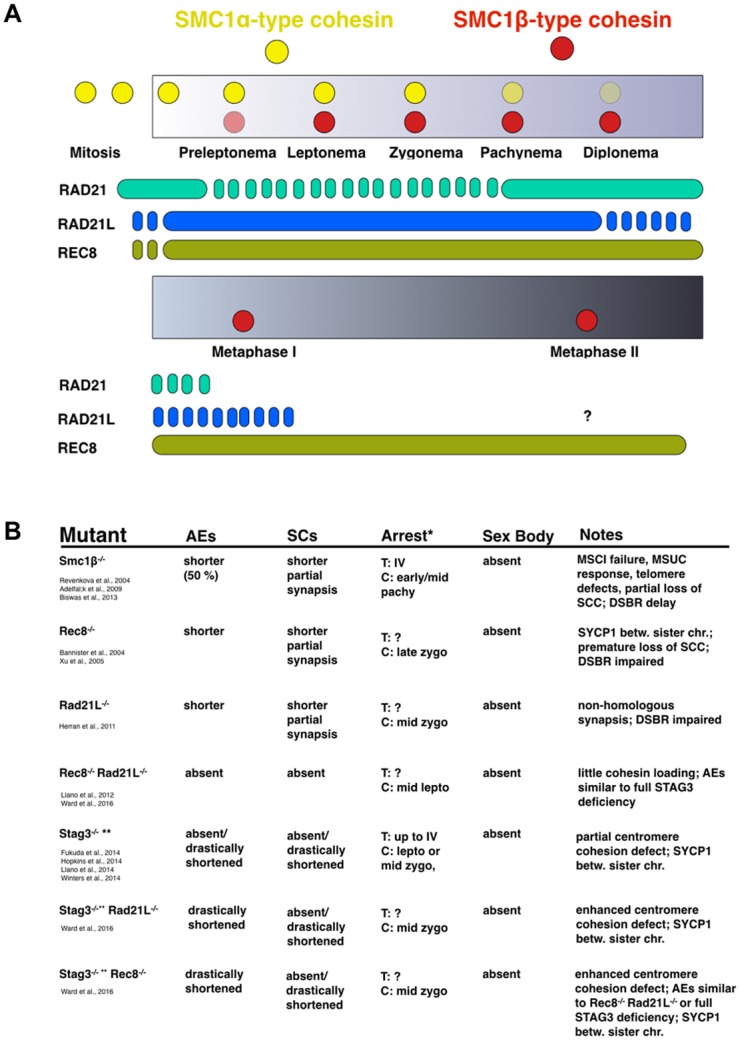
Overview of cohesins in meiosis and meiotic cohesin mutant phenotypes. A. Graph illustrating the approximate occurrence of individual cohesin proteins throughout male meiosis, based on summarizing the current literature; for details see main text. B. Table showing some of the key phenotypes observed in individual mouse strains deficient for one or two cohesin proteins. The respective references are indicated. * T: arrest stage based on most advanced stage of tubular development; C: arrest stage based on appearance of chromosomes and associated proteins; in several instances, the named stages are approximations due to difficulties determining the corresponding normal stage; the mutant stages are often called “-like” stages. ** The *Stag3* mutants used by Hopkins et al, Ward et al., Fukuda et al., and Llano et al., were of distinct origin and express low levels of STAG3 and thus display a hypomorphic phenotype.

Mammalian meiocytes express four meiosis-specific subunits of the core cohesin complex in addition to the ubiquitously expressed five cohesin proteins SMC1α, SMC3, RAD21, SA1/STAG1 or SA2/STAG2. The meiosis-specific cohesins include one SMC protein, SMC1β, the two kleisins RAD21L and REC8, and a stromal antigen protein, SA3/STAG3 (for recent reviews see [2–5]. Theoretically, 18 distinct protein complex can be formed from combinations of these proteins, and so far, at least 6 different cohesin complexes were reported [6–16]. The spatiotemporal appearance of these complexes and their individual roles throughout meiosis are incompletely understood. Immunofluorescence (IF) data derived mainly from staining mouse testis sections or spermatocyte or oocyte chromosome spreads from different stages of prophase I showed distinct patterns of individual cohesin proteins indicating different roles for the various cohesin complexes. The scheme in Fig 1A roughly illustrates the kinetics of presence of individual cohesin proteins in mouse spermatocytes.

In mice of both sexes, SMC1α, SMC1β, and SMC3 are associated with unsynapsed (not yet synapsed), synapsed and desynapsed regions in all stages of prophase I. SMC1α is gradually lost from the chromosomes in diplonema and not detected in metaphase I. SMC1β and much of SMC3 remain associated with the centromeric region until metaphase II. STAG3 behaves similarly to SMC3, and the three kleisins show distinct patterns, which have not yet been entirely clarified as the reports do not agree on all details. Based on imaging studies of spermatocytes it seems as if RAD21 disappears early in prophase I and reappears for a short period in mid to late pachynema and diplonema. REC8 is first seen in preleptotene cells, probably at the onset of premeiotic replication, associates initially all along the spermatocyte chromosomes and remains on centromeres up to metaphase II. RAD21L becomes detectable on chromosomes in leptonema when they start forming AEs, and vanishes at around mid-pachynema. Prior to synapsis, REC8 and RAD21L were observed in a mutually exclusive pattern on the chromosomal axes [8, 9, 13, 15].

Mouse mutants deficient in individual cohesins have revealed very important aspects of their roles. Fig 1B provides an overview of some of the most relevant phenotypes of these cohesin mutants. SMC1β deficient male and female mice are infertile, male meiocytes arrest at a stage when chromosomes have reached an early/mid pachynema structure. With respect to the developmental stage within the seminiferous tubules the cells reached stage IV. It is important to distinguish between the stage of development reached within a section of the seminiferous tubules, and the chromosome features characteristic for a certain stage of meiosis. While in mouse mutants the tubular development may reach a certain more advanced stage, the cells may show chromosome features that are reminiscent of an earlier stage. In other words: the tubules may develop further even though the cells are delayed or blocked in forming the corresponding chromosome structure. Therefore one needs to differentiate between the testis tubule stage and the “chromosomal stage”. Partial loss of cohesion, partial asynapsis, telomere deficiencies, and AEs/SCs that are shortend in length by about half are the prominent phenotypes observed in *Smc1β*^*-/-*^ spermatocytes [17, 18]. In the absence of REC8 both sexes are sterile, the spermatocytes arrest in a late zygonema-like stage based on their chromosomal appearance. Here, synapsis protein SYCP1 is deposited between sister chromatids instead of between homologs [19, 20]. RAD21L deficient spermatocytes do not properly form AEs and synapsis between homologs is abrogated. Spermatogenesis arrests in a zygonema-like chromosomal stage and the males are sterile, whereas females develop age-related infertility [21]. REC8 and RAD21L double deficient spermatocytes are devoid of AEs and SCs and arrest in a leptonema-like chromosomal stage, defined based on the absence of AEs [22]. A similar dramatic phenotype was recently demonstrated for STAG3 deficient mice. Their spermatocytes feature no or–in case of residual low levels of STAG3 proteins–very short AEs, fail in synapsis, lose some centromeric and telomeric sister chromatid cohesion and are sterile [11, 12, 14, 16]. In the complete STAG3 deficiency, the cells develop maximally to a testis tubular stage IV, but chromosomally they reflect leptotene cells as there are no axes (Winters et al., 2014). Very recently, double mutants of Stag3 with either Rec8 or Rad21L were described and show increased centromeric cohesion defects, very short AEs and, in case of the *Stag3*^*-/-*^*Rec8*^*-/-*^ strain, are similar in phenotype to the *Rec8*^*-/-*^*Rad21L*^*-/-*^ spermatocytes [23].

The different kind and/or severity of the phenotypes of mutants in distinct cohesin proteins indicates that specific cohesin complexes contribute during spermatogenesis to distinct processes, which only partially overlap. The functional complexity of the concert of cohesin complexes in meiocytes, however, is far from being sufficiently understood. To further decipher the function of specific meiotic cohesin complexes in male meiosis, we investigated the roles of meiosis-specific kleisins together with the only meiosis-specific SMC protein, SMC1β. Mouse strains carrying deficiencies in SMC1β and either the REC8 or the RAD21L kleisin were generated. The analysis of these double mutants allowed us to determine whether the kleisins act in an SMC1β-based complex. When there were additive effects of double-deficiencies, this would indicate functions of the kleisins in a separate complex, which must be an SMC1α complex. Indeed, we suggest synergistic action of SMC1α and SMC1β complexes to establish proper AE length, synapsis and to maintain telomere integrity. Both meiotic kleisins act together with the two SMC1 variants in these roles. Very early in meiosis I, i.e. in leptonema, the meiosis-specific cohesins are not strictly required for centromeric cohesion.

## Results and Discussion

To assess the contribution of the meiosis-specific cohesin SMC1β in association with either REC8 or RAD21L, we generated *Smc1β*^*-/-*^*Rec8*^*-/-*^ and *Smc1β*^*-/-*^*Rad21L*^*-/-*^ mouse strains. Like the single mutants, the "double knockouts" (DKOs) were sterile and only featured prophase I spermatocytes.

**Fig 2:**
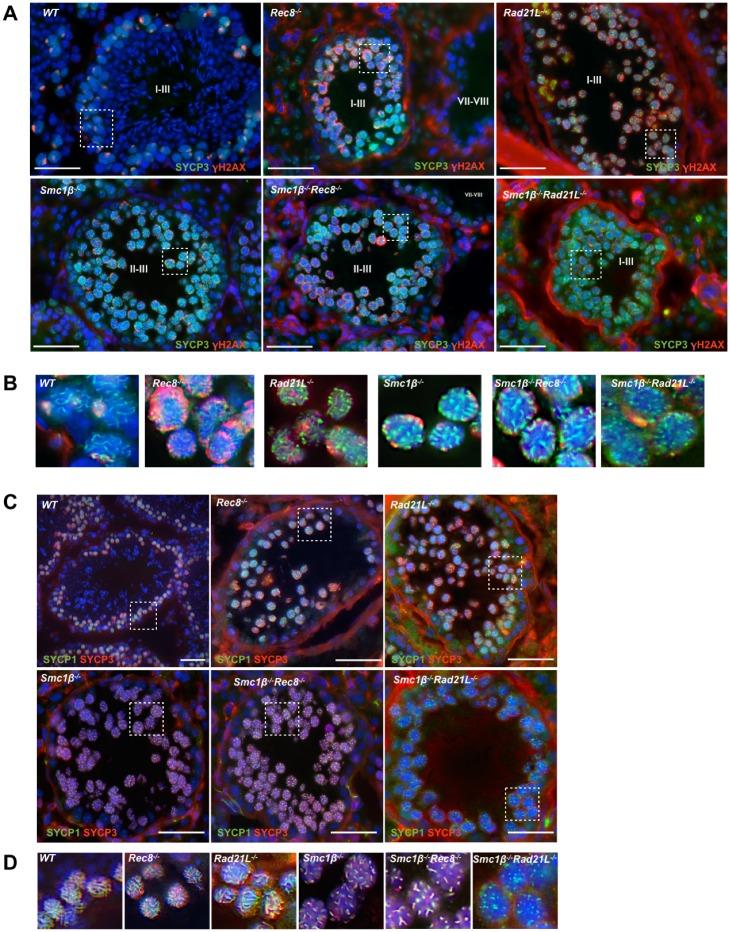
Testis tubule analysis of wild-type and mutant mice. A. Immunofluorescence staining of testis sections of WT, SKO (single knockout) mice *Smc1β*^*-/-*^, *Rec8*^*-/-*^, *Rad21L*^*-/-*^, and DKO (double knock out) mice: *Smc1β*^*-/-*^*Rec8-/-* and *Smc1β*^*-/-*^*Rad21L*^*-/-*^ mice, probed with anti-SYCP3 and anti-γH2AX; DNA was stained with DAPI (scale bar: 5 μm). Tubular stages are indicated by roman letters. B. Magnified images from Fig. 2A showing SYCP3-stained axes and γH2AX localization. The most advanced stages are shown, characterized by chromosome axes that are SYCP3-positive and γH2AX signals, which indicate the presence of DNA double-strand breaks und unsynapsed chromosomes. C. Immunofluorescence staining of testis sections of WT, SKO (single knockout) mice *Smc1β*^*-/-*^, *Rec8*^*-/-*^, *Rad21L*^*-/-*^, and DKO (double-knock out) mice: *Smc1β*^*-/-*^*Rec8-/-* and *Smc1β*^*-/-*^*Rad21L*^*-/-*^ mice, probed with anti-SYCP1 and anti-SYCP3; DNA was stained with DAPI (scale bar: 5 μm). D. Magnified images from Fig. 2C showing SYCP1 (green)/SYCP3 (red)-stained axes indicated by yellow color.

Analysis of testis sections from adult *Smc1β*^*-/-*^*Rec8*^*-/-*^ and *Smc1β*^*-/-*^*Rad21L*^*-/-*^ mice was performed by staining with DAPI, an antibody specific for SYCP3 (“anti SYCP3”), which is a component of the AEs and LEs, and either anti γH2AX or anti SYCP1 (Fig 2A–2D).

**Fig 3:**
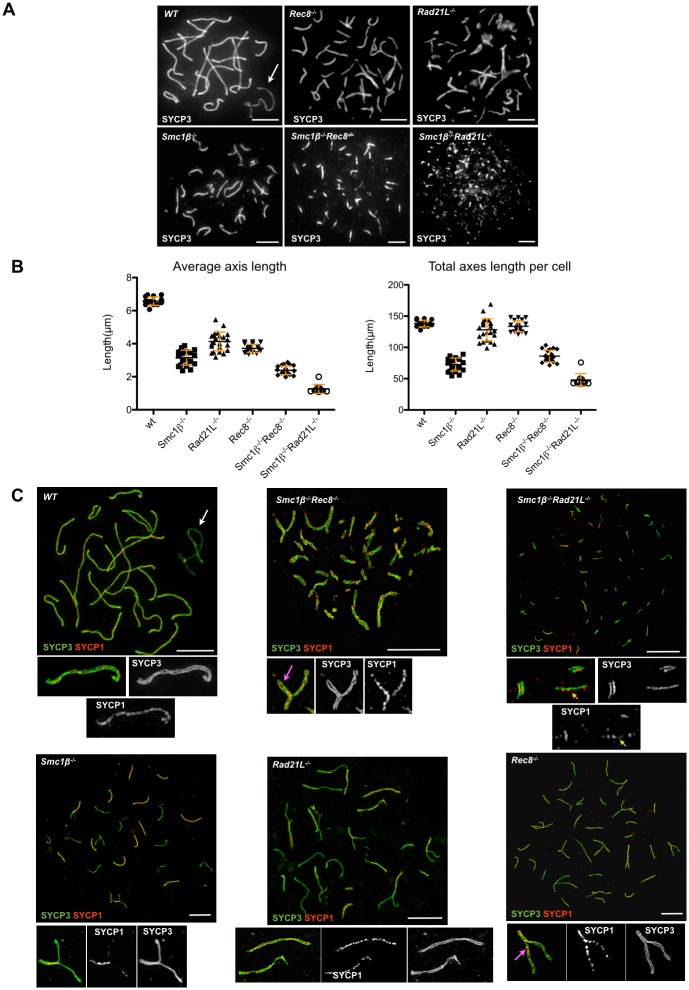
Axes structure and length measurements. A. Immunofluorescence staining of spermatocyte chromosome spreads of WT, SKO and DKO mice, probed with anti-SYCP3 for AEs/LEs. The associated pair of X/Y chromosomes is indicated by a white arrow and present only in WT cells (scale bar: 5 μm). B. Left: Average length (μm) of single chromosome axes per WT, SKO and DKO spermatocyte spreads, measured using the ImageJ software (N = 20, *Smc1β*^*-/-*^; N = 19, *Rec8*^*-/-*^; N = 23, *Rad21L*^*-/-*^; N = 20, *Smc1β*^*-/-*^ *Rec8*^*-/*-^; N = 10, *Smc1β*^*-/-*^ *Rad21L*^*-/-*^); red bars indicate SD. The p values for all comparisons of a mutant to wt are <0.0001. The p values for all other paired comparisons (e.g. *Smc1β*^*-/-*^ versus *Rec8*^*-/-*,^ etc.) are also <0.0001 according to the Mann-Whitney test. Right: average total axes length per cell, i.e. the sum of all axes (μm). This included synapsed and unsynapsed axes, but partially unsynapsed chromosomes were counted as one axis and measured accordingly. High numbers of total axes length per cell despite a reduction of average individual axis length indicates an increase in the number of axes and thus asynapsis (since cohesion was not deficient). Red bars indicate SD. All pairwise differences were statistically relevant (p < 0.05) except for the comparison of wt versus *Rad21L*^*-/-*^, *Smc1β*^*-/-*^ versus *Rec8*^*-/*-^, *Smc1β*^*-/-*^ versus *Smc1β*^*-/-*^ *Rec8*^*-/*-^, *Rad21L*^*-/-*^ versus *Rec8*^*-/*-^ and *Smc1β*^*-/-*^ *Rec8*^*-/*^ versus *Smc1β*^*-/-*^ *Rad21L*^*-/-*^. C. Super resolution (SIM) images of wt, SKO and DKO mutants showing axial elements (SYCP3 positive) and synaptonemal complex (SYCP1 positive). The X/Y chromosomes are indicated by a white arrow, the yellow arrow indicates SYCP1 deposition on a single chromatid, the magenta arrow indicates deposition of SYCP1 between sister chromatids (scale bar: 5 μm).

SYCP1 is a central element protein of the SC and thus a marker for synapsis, γH2AX associates with unsynapsed chromosomes and DSBs. This analysis revealed that the most advanced tubular stage that is completed is stage II-III in both double-mutants, i.e. stages beyond III lack the corresponding pachytene cells. Thus, based on this tubular staging spermatogenesis arrests in very early pachynema. The chromosome structure indicates that some synapsis or at least some potentially irregular deposition of the SC protein SYCP1 occurs. This interpretation is based on the presence of AEs, although shortened, and the presence of at least some SYCP1-positive axes, which in images of *Smc1β*^*-/-*^*Rad21L*^*-/-*^ spermatocytes were almost reduced to dots (Fig 2B and 2D). This suggests a mid zygonema-like chromosomal stage for the most advanced *Smc1β*^*-/-*^*Rec8*^*-/-*^ and *Smc1β*^*-/-*^*Rad21L*^*-/-*^ spermatocytes (see also Fig 3; S1B and S1C Fig).

### Axial element formation in absence of SMC1*β*/REC8 or SMC1*β*/RAD21L

An earlier report showed that the third kleisin RAD21 contributes little or not at all to the formation of AEs in spermatocytes, since there are extremely short SYCP3-positive AEs when the other two kleisins were absent, i.e. in *Rad21L*^*-/-*^*Rec8*^*-/-*^ spermatocytes [10]. At least in this mutant background and for this central function, RAD21 cannot compensate for the loss of the two meiosis-specific kleisins. Thus, it appeared as if the majority of cohesin complexes in prophase I are based on either RAD21L or REC8. Here, we used single and double mutants lacking either REC8 or RAD21L only, or in combination with the SMC1^®^ deficiency to dissect the individual contributions of these cohesins to AE formation. Staining for SYCP3 was used to measure axis length at the most advanced spermatocyte stage in each "single-knockout" (SKO) and *Smc1β*^*-/-*^*Rec8*^*-/-*^ and *Smc1β*^*-/-*^*Rad21L*^*-/-*^ DKOs compared to wild type (wt) (Fig 3A and 3B).

The most advanced stage was assigned based on the appearance and extent of SYCP1 and γH2AX staining, where SYCP1 was present on some chromosomes and the previously diffuse γH2AX signal was reduced to one or two cloud-like structures (S1A and S1B Fig). We took into consideration that some short axes may represent fragments of the same chromosomes and therefore divided the total axes length of a cell by the normal number of chromosomes (21 including X and Y separately). In case of asynapsis of entire chromosomes the number of axes was increased, and we divided the total axes length by this increased number of axes, since each individual axes–whether synapsed or not–was added to the total axes length of a cell. Asynapsis of entire chromosomes was determined by counting the number of CENP-A signals for centromeres. This number was the same in all mutants at the leptotene stage (see below), and if increased at later stages the CENP-A signals on separate axes indicated asynapsis or loss of sister chromatid cohesion and thus an increased number of axes. The total axes length per cell (Fig 3B) was divided by the number of CENP-A positive chromosome axes.

In the most advanced spermatocytes of the *Smc1β*^*-/-*^*Rec8*^*-/-*^ mutant, the axes are very short with an average axis length of 2.351 +/- 0.262 (SD) μm and thus shorter than the corresponding SKO or wt (wt: 6.56 +/- 0.253 μm; *Smc1β*^*-/-*^: 3.27 +/- 0.451 μm; *Rec8*^*-/-*^: 3.67 +/- 0.247 μm; Fig 3A and 3B). Thus, the removal of REC8 in addition to SMC1β further reduces axis length. Therefore, an SMC1α/REC8 complex should exist, unless one would propose a very distinct role of REC8 independent of any cohesin complex, which is very unlikely. The removal of this SMC1α/REC8 complex supposedly accounts for the additional length reduction in this mutant background. Based on the analysis of the *Smc1β*^*-/-*^*Rec8*^*-/-*^ mutant, this SMC1α/REC8 complex contributes a moderate roughly 14% to axis length (i.e. the further reduction by 0.92 μm seen in the *Smc1β*^*-/-*^*Rec8*^*-/-*^ mutant compared to the *Smc1β*^*-/-*^ mutant). Such numbers are obviously approximations only for a normal cell as they reflect the contributions in a mutant background. At any rate, this SMC1α/REC8 complex appears to be a minor complex. This is in agreement with the low efficiency or absence of co-precipitation of SMC1α and REC8 from wt or *Smc1β*^*-/-*^ testis extracts reported earlier [9, 13, 17].

Despite the moderate reduction is axis length in *Rec8*^*-/-*^ and *Rad21L*^*-/-*^ cells, the total axes length per cell is similar to wt (Fig 3B). This originates from the high levels of asynapsis in these mutants.

From the almost total reduction of axis length in STAG3 deficient spermatocytes [16] it is clear that cohesins determine the entire axis length. In the *Smc1β*^*-/-*^*Rec8*^*-/-*^ mutant with only 2.35 μm of axis length left, these remaining axis–app. 36% of wt length only–must also be provided by some cohesin complex(es). Thus, the remaining app. 36% of axis length that still exists in *Smc1β*^*-/-*^*Rec8*^-/-^ spermatocytes has to be supported either by SMC1α/RAD21 or SMC1α/RAD21L complexes, the only remaining complexes.

Due to non-homologous associations of AEs and to gaps in SYCP3-positive AEs, the measurement of axis length in *Rad21L*^*-/-*^ spermatocytes is very difficult, but an estimate that only takes non-associated, gap-less and clearly identifiable axes into account yields a length roughly comparable to that of the *Rec8*^*-/-*^ strain (Fig 3B). In *Smc1β*^*-/-*^*Rad21L*^*-/-*^ spermatocyte spreads we observed very short SYCP3-stained axes, which often appeared as dots rather than as filaments; they measured 1.17 +/- 0.27 μm (Fig 3A and 3B).

Thus, in contrast to the *Rad21L*^*-/-*^ or *Smc1β*^*-/-*^ SKOs, the removal of SMC1β and RAD21L almost entirely abolishes formation of SYCP3-positive axes, with no obvious compensatory effect. This suggests that besides SMC1β complexes, an SMC1α/RAD21L complex contributes to axis formation. This further suggests that an SMC1α/RAD21 complex contributes little if any to axes length. In at least one report anti SMC1α precipitation co-precipitated RAD21L [13]. Since the combined loss of RAD21L and REC8 also causes almost complete loss of axes [10], this supports the above notion that an SMC1α/RAD21 complex does not significantly contribute to axis length and RAD21 cannot compensate for the loss of the two other kleisins, at least under conditions where other cohesins are absent. Similarly, since the *Smc1β*^*-/-*^*Rad21L*^*-/-*^ and the *Rec8*^-/-^*Rad21L*^*-/-*^ [10] DKOs essentially abolish axis formation, but the *Smc1β*^*-/-*^, *the Rec8*^-/-^ and the *Rad21L*^*-/-*^ SKOs do not, the SMC1α/RAD21L and the SMC1β/REC8 complexes must very prominently if not almost entirely support axis formation, with the above mentioned minor contribution of SMC1α/REC8.

A potential role of RAD21 should therefore be mostly confined to other, specific functions such as supporting pachynema/diplonema events, perhaps formation of chiasmata, consistent with the reappearance of RAD21 seen in some studies at this stage.

It should be noted that all numbers provided here as percentage of contribution to axis length are based on comparison with the wt situation. In any mutant, compensatory mechanisms may arise that may affect these numbers such as increased expression or stability of the remaining cohesin complexes. Thus, conclusions are qualitative and only roughly quantitative. However, any compensatory effect, if it exists, may be very minor, since axes are reduced to almost dots in the *Smc1β*^*-/-*^*Rad21L*^*-/-*^ spermatocytes, and a very similar observation has been made in a STAG3 deficient mutant–no rescue by other STAG proteins [16]. Therefore, the numbers suggest the relative importance of particular complexes to axis length not only in the specific mutant backgrounds, but very likely also with respect to wt cells. In any case, the analysis reveals the presence and functional capacities of certain cohesin complexes in spermatocytes.

### Synapsis and DNA double-strand breaks

Synapsis is impaired in all mutants as co-staining for SYCP1 and SYCP3 showed (S1B and S1C Fig). However, it is not possible to precisely quantify the extent of synapsis for the individual mutants, since in absence of REC8 the SYCP1 deposits between sister chromatids yielding a “false” signal, and in the *Smc1β*^*-/-*^*Rad21L*^-/-^ mutant very small axes or dots appear. Many (app. 70%) of these extremely small structures carry SYCP1, but the small size precludes quantification. Co-staining for HORMAD1, an asynapsis marker, and SYCP3 confirmed the extent of synapsis failure in all the mutants, since HORMAD1 is present in all cases (S1D Fig).

Detailed analyses of SYCP3- and SYCP1-stained axes of the wt and DKO spermatocytes by super-high resolution OMX microscopy (Fig 3C) showed the expected central localization of SYCP1 between the two SYCP3 axes in wt cells. In the *Smc1β*^*-/-*^*Rec8*^-/-^ mutant, where no synapsis occurs and the SYCP3-stained axes therefore consist of two sister chromatids, the deposition of SYCP1 between the sister chromatids is clearly observed and is in agreement with previous reports that described this phenotype for the *Rec8*^-/-^ strain [19, 20]. In the *Smc1β*^*-/-*^*Rad21L*^-/-^ mutant, SYCP1 is also deposited between sister chromatids, but in addition some individual SYCP1-positive sister chromatids were observed, typically 7 per cell (+/- 5.0; n = 40). This data indicates moderate loss of sister chromatid cohesion in this mutant and show that SYCP1 does neither require two sister chromatids in cohesion nor chromatids in SYCP3-mediated close proximity, nor two AEs, to associate along chromosomes. The deposition of SYCP1 at a single chromatid also suggests, that in mutant backgrounds SYCP1 deposition is not necessarily an indicator for synapsis. Therefore we prefer not to designate SYCP1 deposition on pairs of sister chromatids as "synapsis between sister chromatids".

In wt mouse spermatocytes, the sex chromosomes X and Y only pair at a short, centromere-distal region called the pseudo-autosomal region, PAR [24]. The largely unsynapsed sex chromosomes form a special chromatin domain, the sex body, which features silencer chromatin marks. This X/Y association is seen only in wt cells (Fig 3A, arrow), as is the characteristic sex body chromatin staining by γH2AX as one intense structure with the sex chromosomes embedded (S1A Fig, arrow).

**Fig 4:**
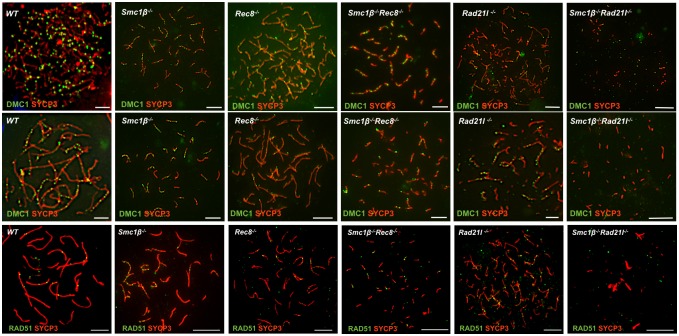
DNA double strand break repair foci. Immunofluorescence staining of spermatocyte chromosome spreads of WT, SKO and DKO mice, probed with anti-SYCP3 (red) for AEs/LEs and anti-DMC1 (green) or anti RAD51 as indicated for DNA double-strand break repair foci. Upper panel: leptotene/early zygotene stage with many DMC1 foci; middle panel: late zygotene/early pachytene like stage with reduced number of DMC1 foci; lower panel: RAD51 foci on pachytene-stage like cells (scale bar: 5 μm).

Synapsis of homologs depends on programmed double-strand breaks (DSBs), which are repaired with progressing synapsis. Cohesin SMC1β is not required for generation of DSBs, but was shown to support their repair, which is delayed in absence of SMC1β [25]. DSBs can be visualized by staining of DSB repair proteins such as RAD51 or the meiosis-specific DMC1. In all mutants reported here, DMC1 foci and thus DSBs are produced (Fig 4).

Quantification of DMC1 in the DKOs is very difficult as the axes are short or only dots exist where one cannot distinguish individual foci, particularly in the more advanced stages. Therefore we cannot provide exact numbers. The initial numbers of DMC1 foci, as much as recognizable, appeared to be very similar in the mutants and not unlike wt. The same was observed for RAD51 foci. In the more advanced stage of *Smc1β*^*-/-*^*Rec8*^*-/-*^ cells the number of DMC1 foci is reduced to two or more foci per short axis. A similar reduction was observed in *Rec8*^*-/-*^ spermatocytes but not in *Smc1β*^*-/-*^ spermatocytes, where repair is delayed as previously reported [25]. One may speculate that in the *Smc1β*^*-/-*^*Rec8*^*-/-*^ spermatocytes alternative repair pathways such as between sister chromatids supported by SYCP1 localized between sister chromatids are enhanced or that the foci are less stable. We also found several discrete DMC1 foci in *Smc1β*^*-/-*^*Rad21L*^*-/-*^ spermatocytes, but due to the extremely short axes we cannot clearly distinguish foci. We observed a reduction of DMC1 signals in *Smc1β*^*-/-*^*Rad21L*^*-/-*^ spermatocytes in the advanced stage, indicating that repair of DSBs happens or that the foci are not stable.

### Centromeric cohesion

The analysis of centromeres of meiotic chromosomes reveals both, synapsis at centromeres and for centromeric sister chromatid cohesion. In wt pachynema spermatocytes 21 centromere signals indicate complete synapsis (except the sex chromosomes with their PAR-distal and thus non-synapsed centromeres) and complete centromeric sister chromatid cohesion. The appearance of 22 to 40 centromere signals indicates either loss of synapsis, or loss of centromeric cohesion in presence of full synapsis, or partial loss of both cohesion and synapsis. More than 40 centromere signals indicate loss of some or all (80 signals) centromeric cohesion and synapsis. In early zygotene cells, where no synapsis exists and thus this analysis of cohesion is less perturbed by synapsis, more than 40 centromere signals (from 40 AEs, i.e. 2 x 19 autosomes plus X and Y) indicate loss of centromeric cohesion. Since the DKOs develop to an early/mid zygonema-like chromosomal stage, we analyzed all cells at the leptotene/early zygotene stage to be able to test for centromeric cohesion independent of synapsis, i.e. we expect 40 centromere signals in wt cells.

**Fig 5:**
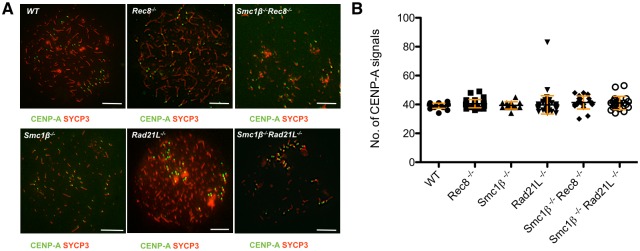
Centromeric cohesion. A. Immunofluorescence staining of spermatocyte chromosome spreads of leptotene stage of WT, SKO and DKO mice, probed with anti-SYCP3 for AEs/LEs and anti-CENP-A for the inner kinetochore (scale bar: 5 μm). CENP-A signals near the ends of chromosomes mark the centromeres and were counted. B. Graph showing the average number of CENP-A signals of WT, SKO leptotene/early zygotene spermatocytes, indicative of centromeric cohesion. (N = 14, Smc1β^-/-^; N = 34, Rec8^-/-^; N = 59, Rad21L^-/-^; N = 21, Smc1β^-/-^ Rec8^-/-^; N = 22, Smc1β^-/-^ Rad21L^-/-^); red bars indicate SD. There was no significant difference between any wt or mutant strain (all p vales >0.04; Dunn’s multiple comparison test).

Previously it has been shown that depletion of REC8 causes synapsis failure. However, REC8 is not required for the establishment of centromeric sister chromatid cohesion in meiocytes as in the most advanced *Rec8*^*-/-*^ spermatocytes never more that 40 centromeres were observed [19]. We also observed in average 37.76 (+/- 1.787, n = 42) centromeres in *Rec8*^*-/-*^ spermatocytes by staining with anti-centromeric antibodies (ACA) (S2 Fig), which recognize centromeric and pericentromeric heterochromatin. Staining for CENP-A, an inner centromere component showed 40.5 (+/- 2.84; n = 34) signals in leptonema *Rec8*^*-/-*^ spermatocytes (Fig 5A and 5B).

This indicates proper centromeric cohesion despite absence of REC8. In *Smc1β*^*-/-*^*Rec8*^*-/-*^ spermatocytes we observed an average of 36.12 (+/- 5.930; n = 33) ACA signals, and 41.4 (+/- 4.5; n = 21) CENP-A signals (Fig 5A and 5B; S2 Fig), again suggesting maintenance of centromeric cohesion, although the very slight increase in CENP-A signals may hint at a minor trend towards weakening of centromeric cohesion. The situation is similar in the other mutants: *Rad21L*^*-/-*^ spermatocyte spreads showed 30.44 (+/- 4.330; n = 52) ACA and 39.8 (+/- 6.4; n = 59) CENP-A signals on average in accordance with a previous report that used ACA [21]. The low number of distinguishable ACA signals may be caused by telomere fusions (see below), which can bring the relatively broad centromeric heterochromatin in very close proximity. CENP-A, which provides a more specific signal just at the inner kinetochore, would not be as much affected. *Smc1β*^*-/-*^*Rad21L*^*-/-*^ spermatocytes showed 39.09 (+/- 5.39; n = 46) ACA signals and 41.0 (+/- 4.6; n = 22) CENP-A signals. Statistically significant differences were not found for CENP-A signals, and only some differences between certain pairs of mutants were statistically different for the ACA signals with a p-value >0.05. Overall there appears to be slightly more variation in centromere numbers in the DKOs, but this suggests very limited if any weakening of centromeric cohesion.

This data indicates that none of the three meiotic cohesins analyzed here are required for early prophase cohesion. Thus, an SMC1α complex, i.e. SMC1α/RAD21, must provide most if not all centromeric cohesion at this very early stage of male meiosis. This is consistent with the notion that SMC1*β* is only expressed after entry into meiosis [7], and is in agreement with the only partial loss of centromeric cohesion in okadaic acid-induced metaphase I *Smc1β*^*-/-*^ spermatocytes when these were derived from zygonema cells. When the cells originated from early/mid pachynema, complete loss of centromeric cohesion was observed [17]. This suggests that SMC1β complexes are loaded onto meiotic chromosomes during prophase I, or at least become cohesive then, and successively take over the duty of maintaining centromeric cohesion from the SMC1α/RAD21 complex, which progressively vanishes after entry into meiosis.

How can one reconcile this with our analysis of *Smc1β*^*-/-*^*Spo11*^*-/-*^ and *Smc1β*^*-/-*^ spermatocytes, which showed some centromeric cohesion deficiency in absence of SMC1β [25]? In that study, spermatocytes of the most advanced stages were analyzed, i.e. late zygonema for *Smc1β*^*-/-*^*Spo11*^*-/-*^ or early/mid pachynema for *Smc1β*^*-/-*^. At this stage, partially synapsed axes carrying three separate centromeres were observed in *Smc1β*^*-/-*^ cells, which clearly indicate both, synapsis failure combined with loss of cohesion at the centromeres in at least 8% of the cases. If centromeric synapsis fails, the one strong signal derived from 4 very closely juxtaposed centromeres falls apart into two signals; 3 or 4 signals then indicate loss of cohesion. In *Smc1β*^*-/-*^*Spo11*^*-/-*^ chromosomes, which do not synapse, one or two ACA signals were observed and indicated loss of cohesion in about a third of the cells. We suggest that while at leptonema the SMC1α/RAD21 complex still provides most centromeric cohesion, it is partly replaced by SMC1β complexes when synapsis starts to happen, i.e. in zygonema. This interpretation would fit to the above notion of loading of SMC1β complexes onto centromeres after entry into meiotic prophase I.

### Presence of cohesin proteins on mutant spermatocyte chromosomes

In the absence of SMC1β, SMC1α and SMC3 localize to AEs of early prophase I cells [17] suggesting that the SMC1〈/SMC3 heterodimer forms complexes with RAD21L, REC8 or RAD21 or either of them *in vivo*. Evidence from immuno precipitation experiments differs somewhat between distinct reports [9, 13, 15, 17], but neither of the three complexes can be surely excluded at this time.

**Fig 6:**
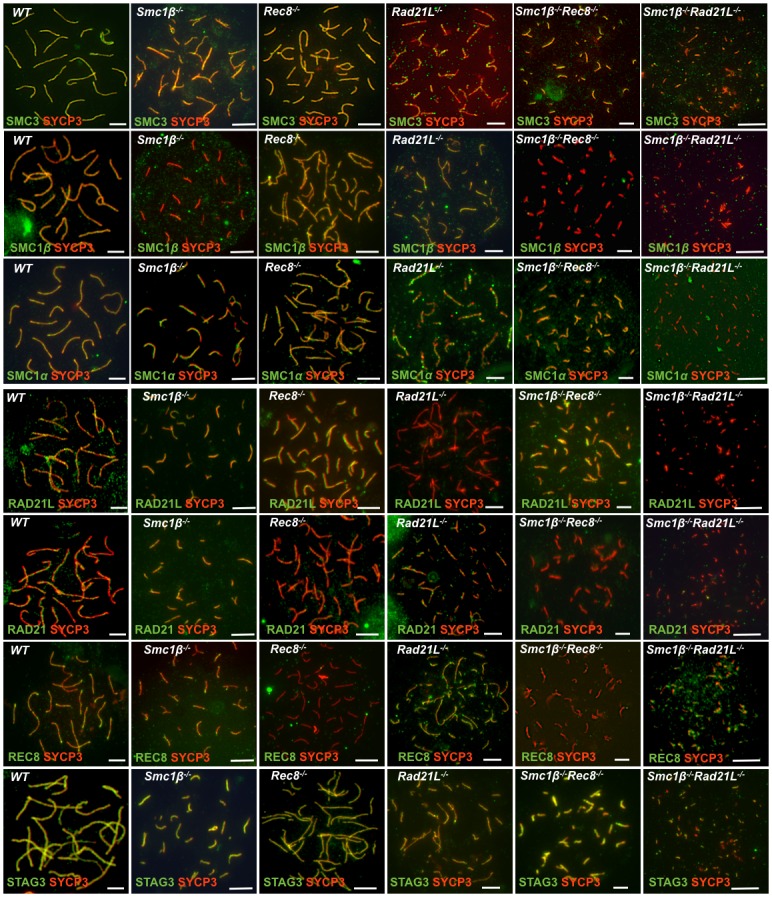
Localization of cohesin proteins. Immunofluorescence staining of spermatocyte chromosome spreads of WT, SKO and DKO mice probed with anti-SYCP3, anti-SMC3, anti-SMC1β, anti-SMC1α, anti-RAD21L, anti-RAD21, anti-STAG3 or anti-REC8 (scale bar: 5 μm). All cohesins are labeled green and show either rather uniformly along the chromosomes or in a dot-like pattern. The single channel images provided in S4–S10 Figs may allow a more precise observation of staining patterns. Please note that while the comparison of mutants and WT stained with one particular antibody is possible, the interpretation of comparisons of staining patterns of different antibodies had to be made with much caution, since because antibody affinities and other features differ.

We analyzed chromosomal localization of the remaining cohesin proteins in *Smc1β*^*-/-*^*Rec8*^*-/-*^ and *Smc1β*^*-/-*^*Rad21L*^*-/-*^ spermatocytes in comparison to wt and the SKOs (Fig 6; for single channel images see S3 to S9 Figs).

The one cohesin present in all known cohesin complexes, SMC3, localizes to the chromosomes in all mutants, although at much reduced amounts in the two DKOs. The pattern also becomes more dotty, much less uniform in the DKOs and also in *Rad21L*^*-/-*^ than in wt cells. Whether the occasional signals located off the axes are true chromatin signals cannot be ascertained, although such signals rarely appear outside the nuclei; still at least some can be unspecific signals derived from sticky chromatin. The presence of SMC3 in the DKOs indicates that there are SMC1α complexes, either with RAD21 and/or with either RAD21L or REC8. This is consistent with the notion above on the role of SMC1α complexes in cohesion and axis length. SMC1β is present in REC8 and RAD21L deficient cells, indicative of SMC1β in association with either one or two of the other kleisins that are present in these mutants. SMC1α is present along the chromosome axes in all SKOs, as well as in the *Smc1β*^*-/-*^*Rec8*^*-/-*^ chromosome spreads. A somewhat weaker signal was observed in the *Rad21L*^*-/-*^ spreads. This may indicate the disappearance of an SMC1αRAD21L complex. The *Smc1β*^*-/-*^*Rad21L*^*-/-*^ preparations show the weakest SMC1α signal, if any. In most cells no specific SMC1α signal was observed. Since the removal of SMC1β in addition to RAD21L should, in principle, not affect SMC1α that is not associated with RAD21L, the nearly complete absence of SMC1α is not immediately intuitive. We think that the absence of a discernible signal may be due to the extremely short, dot-like axes, which would not allow SMC1α to efficiently associate with them. Thus, the SMC3 and SMC1α patterns are largely consistent with each other, although different antibodies generate different intensities, which therefore can hardly be directly compared.

Similarly, RAD21L is present in all spreads except those of *Rad21L*^*-/-*^ and *Smc1β*^*-/-*^*Rad21L*^*-/-*^ mice. Widespread signals for REC8 are seen in the *Rad21L*^*-/-*^ spreads, much less is present in *Smc1β*^*-/-*^ and *Smc1β*^*-/-*^*Rad21L*^*-/-*^ samples. In agreement with the above notion of a minor contribution of SMC1α/REC8 complex to axis formation, this suggests that the majority of REC8 complexes are SMC1β based. RAD21 is more chromatin-spread and axes-associated in wt, and there is still some RAD21 on *Smc1β*^*-/-*^ and *Rad21L*^*-/-*^ spermatocyte chromosomes, but less on *Rec8*^*-/-*^ chromosomes. Very little is seen on either of the DKO spreads, likely because these cells do not enter pachynema, the stage when RAD21 would reappear. Together this is in accordance with the notion that SMC1α/RAD21 complexes exist on spermatocyte prophase I chromosomes. Consistent with the finding of normal numbers of centromere signals in all mutants, we found that those other two kleisins that are present in a given kleisin mutant localize to the centromeres.

The only meiosis-specific SA-type cohesin STAG3/SA3 localizes all along the wt and *Smc1β*^*-/-*^ chromosomes indicative of SMC1α/STAG3 complexes as reported before [17]. STAG3 also associates along *Rec8*^*-/-*^ chromosomes and is present at least in a dotty pattern on *Rad21L*^*-/-*^ chromosomes. However, *Smc1β*^*-/-*^*Rec8*^*-/-*^ chromosome spreads show STAG3 signals, which suggest that a complex of SMC1α/STAG3 provides the signals seen on *Smc1β*^*-/-*^ and on DKO chromosomes. The STAG3 signals seen on *Smc1β*^*-/-*^*Rad21L*^*-/-*^ miniature axes are consistent with a SMC1α/STAG3/REC8 complex, which should not be eliminated in this DKO. In immuno precipitation experiments, STAG3 co-precipitated with either of the SMC1 variants, and precipitated with RAD21, RAD21L and REC8 although not in all experiments reported [9, 13, 17, 21].

While obviously the absence of one cohesin does not preclude others to associate with chromosomes, one potential caveat of the analysis is that chromosome association or cohesin expression of one cohesin could be increased if another one is missing. However, the strong phenotypes observed in each mutant clearly indicate that no full, probably not even relevant, compensation by other cohesins exist. Changes in expression levels of other cohesins in a particular single cohesin mutant were not observed so far [9, 13, 17, 21].

### Telomere deficiencies

**Fig 7:**
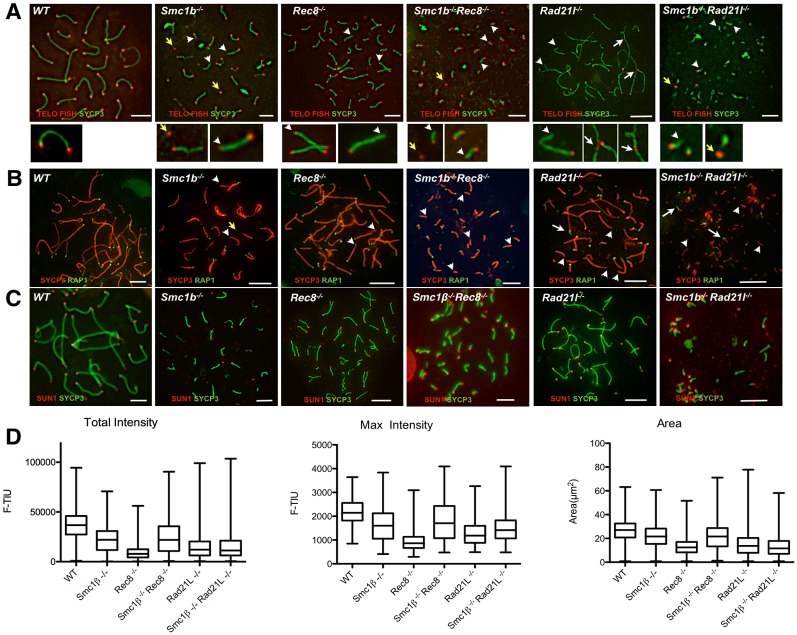
Telomere analysis. A. Staining of spermatocyte spreads by Telo FISH (red) to assess telomere length and anti-SYCP3 for AEs/LEs in WT, SKO and DKO mice (scale bar: 5 μm). Magnified images of individual chromosomes are shown below. Telomeres are seen as red dots at the ends of chromosomes or on disrupted chromosome structures. WT chromosomes show FISH signals of about equal intensities at both ends. Examples for aberrant telomeres are indicated as follows: SCs lacking telomeres—white arrow head; isolated telomeres/broken off SCs, yellow arrow; telomere fusions or close associations—white arrow. B. Immunofluorescence staining of spermatocyte chromosome spreads of WT, SKO and DKO mice, probed with anti-SYCP3 (red) for the AEs/LEs and anti-RAP1 (green) to stain telomeres (scale bar: 5 μm). Aberrant telomeres are marked as above. C. Immunofluorescence staining of spermatocyte chromosome spreads of WT, SKO and DKO mice, probed with anti-SYCP3 (green) for the AEs/LEs and anti-SUN1 (red) to stain telomere attachments (scale bar: 5 μm). D. Graph showing the average telomere intensity, maximum intensity and area of WT, SKO and DKO spermatocyte spreads as measured using the ImageJ software. The boxes show the median value, the upper 75th and the lower 25th percentile, along with maximal and minimal values. The p values for all comparisons of a mutant to wt are <0.0001. The p values for all other paired comparisons (e.g. *Smc1β*^*-/-*^ versus *Rec8*^*-/-*^ etc.) are also <0.0001 according to Dunn’s multiple comparison test.

Earlier we reported telomere deficiencies in *Smc1β*^*-/-*^ spermatocytes [26]. These deficiencies included shortened telomeres, SCs without telomeres, telomeres that have apparently been broken off SCs, and telomere fusions. To test the contribution of individual kleisins to telomere integrity, we stained chromosome spreads of wt and all mutants by FISH for telomeric sequences (telo-FISH), by anti-RAP1 for this telomere-specific protein, and by anti-SUN1 for association of telomeres with the SUN/KASH complex (reviewed in [27]), which anchors telomeres at the nuclear membrane in early prophase I (Fig 7A–7D; S11 Fig).

Because of the very short or dot-like axes of the two DKOs it is not possible to quantify individual telomeres or telomere-like structures, but a qualitative description can be provided. In wt cells, 19 autosomes in full synapsis generate 38 telomere signals, the X and Y chromosomes yield 3 telomere signals, since the synapsed PAR telomere appears as one, together 41 signals. The loss of PAR synapsis in all mutants increases this number to 42. A fully unsynapsed autosome shows 4 signals, if there is additional loss of cohesion, 8 signals emerge. Telomere aberrations such as telo-less axes or solitary telomere fragments perturb these numbers.

**Fig 8:**
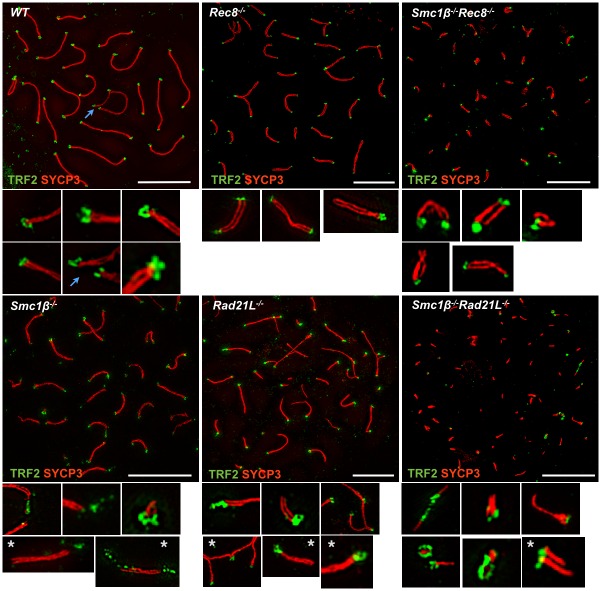
Super resolution telomere analysis. SIM analysis of wild-type and mutant spermatocyte telomeres in chromosome spreads, stained with anti TRF2 and anti SYCP3. Sex chromosomes in wild-type are marked by a blue arrow. High magnification excerpts labeled with a grey asterisks are additional examples from separate images (scale bar: 5 μm).

In all mutants, telo-FISH shows aberrant telomeres with telomeric DNA that seems to have ruptured off the axes, with axes that lack telomere signals, and with telomeric ends tightly associated, perhaps fused, or clustered (Fig 7A). These phenotypes are most prominently seen in SMC1β and RAD21L deficient mutants. *Rec8*^*-/-*^ spermatocytes show the fewest telomeric aberrations, although often 3- to 4 chromosomes display a low intensity FISH signal at one end. FISH signals are directly proportional to telomere length (see below and, for example [26]). RAP1 staining (Fig 7B) confirms that many telomeres are deficient in the DKOs, since many axes lack RAP1 signals. These aberrations were also confirmed by staining for TRF2 (see below; Fig 8 and S12 Fig).

Similarly, the SKO and DKO show reduced number of SUN1 spots (Fig 7C), indicative of failure to associate with the nuclear periphery. We counted the number of SUN1 telomere signals in all the mutants of the most advanced stage. We observed in average 45.32 (+/- 3.787, n = 26) SUN1 signals in wt zygotene/pachytene spermatocytes. In *Rec8*^*-/-*^, *Smc1β*^*-/-*^ and *Rad21L*^*-/-*^ spermatocytes we observed 64.08 (+/- 5.787, n = 25), 43.3 (+/- 2.787, n = 26) and 42.3 (+/- 6.787; n = 26) SUN1 signals, respectively. In *Smc1β*^*-/-*^*Rec8*^*-/-*^ and *Smc1β*^*-/-*^*Rad21L*^*-/-*^ spermatocytes we observed in average only 44.3 (+/- 5.987, n = 23) and 39.3 (+/- 10.587, n = 34) signals (S11 Fig). Differences were statistically significant with a p-value <0.05 for the comparisons of wt versus *Rec8*^*-/*-^, *Smc1β*^*-/-*^ versus *Rec8*^*-/*-^, *Rec8*^*-/*-^ versus *Smc1β*^*-/-*^ *Rec8*^*-/*-^, *Rec8*^*-/*-^ versus *Rad21L*^*-/-*^, and *Rec8*^*-/*-^ versus *Smc1β*^*-/-*^*Rad21L*^*-/-*^. Thus, the only statistically significant difference was observed with the REC8 deficiency. Detailed interpretation of these numbers is difficult as many processes contribute in different ways. Increased SUN1 numbers may result from unsynapsed chromosomes that each form SUN1 foci. Telomere fragments may also form SUN1 foci as seen mostly in *Rad21L*^*-/-*^ cells. Decreased SUN1 foci most likely reflect the loss of telomere ends seen in all mutants, not compensated for by unsynapsed chromosomes. Why, for example, there are fewer SUN1 foci in the *Smc1β*^*-/-*^*Rec8*^*-/-*^ cells than in the *Rec8*^*-/-*^ spermatocytes, which show comparable levels of asynapsis, can only be speculated about: SMC1β may be much more required to preserve telomeric DNA and its structure than REC8, consistent with the many aberrations seen in the *Smc1β*^*-/-*^ spermatocytes [26]. Overall, this data reflects the expected telomere and telomere attachment deficiencies.

Measurements of the intensity of the FISH signal (Fig 7D) showed that in all mutants there is a shift towards lower intensity, which indicates shorter telomeres. Telomere intensities peak in the wt at 35000 to 40000 units. The *Smc1β*^*-/-*^ spermatocytes display a peak around 20000 units and thus feature shorter telomeres as reported before [26]. In *Rec8*^*-/-*^ spermatocytes the median intensity is at app. 7500 units and thus telomeres are even shorter than in *Smc1β*^*-/-*^ spermatocytes. Unexpectedly, *Smc1β*^*-/-*^*Rec8*^*-/-*^ spermatocytes show more intense telomere signals, indicating that other modes of telomere protection become effective if these two cohesins are absent. The median intensity in *Smc1β*^*-/-*^*Rec8*^*-/-*^ spermatocytes is similar to that in *Smc1β*^*-/-*^ spermatocytes, and the difference is not statistically significant. Both however show higher intensity and thus longer telomeres than in *Rec8*^*-/-*^ spermatocytes. This indicates SMC1β is a main contributor to telomere length and it does so without REC8, i.e. in a different complex. The effect of REC8 deficiency can thus only be brought about by an SMC1α/REC8 complex. *Rad21L*^*-/-*^ and *Smc1β*^*-/-*^*Rad21L*^*-/-*^ spermatocytes show very short telomeres, with average intensities peaking at around 10.000, and there is no statistically relevant difference between these two strains. This suggests that RAD21L is mainly associated with SMC1β in this function. The variation in length is particularly extensive in the *Rad21L*^*-/-*^ and *Smc1β*^*-/-*^*Rad21L*^*-/-*^ spermatocytes. Together this suggests that an SMC1β/RAD21L complex and an SMC1α/REC8 complex are mainly responsible for proper telomere length. There is no additive effect of removing RAD21L in addition to SMC1β. This supports the notion that an SMC1α complex featuring RAD21L does not significantly contribute to telomere length.

To reveal ultrastructural features of telomeres we performed super-resolution imaging (SIM) on anti TRF2-stained telomeres of wild-type and mutant spermatocytes (Fig 8, S12 Fig).

The analysis confirmed the presence of telomere aberrations on mutant chromosomes as described above (Fig 8). In addition, we observed loop-like structures on many wild-type telomeres, but rarely on mutant telomeres (Fig 8, S12 Fig). Plotting a 3D-image from signal intensities to analyze contour plots shows a circle of 4 telomere spots in many wt instances (S13 Fig). Quantification of these loop-like structures showed that 64% of the wt, but less than 10% of the mutant chromosomes carry such structures (S14 Fig). Multiple telomere signals were seen in a third of wt samples, but in half or more of the mutants. The mutants often (36 to 48%) also showed only one telomere signal per chromosome, i.e. one end lacked a signal, which happened only in 4% of wt cases. Stretches of telomere signals were observed only in mutants. We assume that almost all wt telomeres feature these loop-like structures, since depending on the specific plane the telomeres were looked at, one may not be able to see all of them as distinct circles, and some may be lost upon chromosome spreading. The non-paired ends of sex chromosomes of wt often also show circles. This suggests that cohesins, particularly SMC1β complexes, support formation of a more closed conformation at the very end of telomeres, which may represent a protective structure. These loops are reminiscent of TRF2-positive t-loops reported from somatic cells [28] and of telomere complexes reported recently for spermatocytes [29].

### Conclusions

In conclusion, the different cohesin complexes that exist in mammalian spermatocytes contribute distinctly to different structures and processes in these cells. S1 Table summarizes the most important observations. Some of our conclusions assume that there is no role of kleisins independently of a cohesin complex, i.e. independently of either SMC1α or SMC1β. Formally this can hardly be excluded, but there is no evidence for this. We think the assumption that kleisins work only within cohesin complexes is very reasonable. So far all known functions of kleisins are consistent with their association with cohesins, and thus the interpretations provided above and below are the most straightforward.

**Table 1:** List of primary antibodies used.

Primary antibody	Antibody isotype	Source	Working concentrations:IF (IB)
SMC1α	Mouse IgG	Jessberger	1:100
RAD51	Rabbit	Santa Cruz(SC-8349)	1:100
DMC1	Rabbit	Santa Cruz(SC-22768)	1:100
RAD21	Rabbit	Bethyl(A300-080A)	1:200
RAD21	Rabbit	Abcam(ab154769)	1:100
RAD21L	Rabbit	Pendas	1:500(1:1000)
STAG3	Rabbit	Jessberger	1:100(1:1000)
SYCP3	Rabbit	Novus Biologicals(NB300-230)	1:500
anti-ACA	human IgG	AntibodiesInc.(15-235-0001)	1:5
antiSMC1b-N (5048)	Mouse IgG	Jessberger	1:100
SMC3	Rabbit	Bethyl(A300-060A)	1:100(1:2000)
γH2AX Ser 139	Mouse IgG1	Upstate(05–636)	1:700
SYCP3 (Klon 60C10)	MouseIgG1 (hybridoma)	Jessberger	undil. supernatant
HORMAD1	Guineapig	Attila Toth	1:700
SYCP1	Rabbit	Abcam(ab15090)	1:500
CENPA	Rabbit	Cell Signalling(20485)	1:100
REC8	Guineapig	Höög	1:100(1:1000)
SUN1	Guineapig	Alsheimer	1:500

Several complexes contribute to axes formation and define their length, but to different extent. As determined in mutant backgrounds, SMC1β complexes determine about half of axes length, SMC1α complexes provide the other half with an SMC1α/RAD21L complex supporting axes length most prominently with roughly one-third, the SMC1α/REC8 complex only contributes a minor fraction. A significant contribution by RAD21 complexes is unlikely. The additive effect of distinct cohesin complexes to axes length suggests that the amount of cohesin available to be loaded onto meiotic chromosomes determines axes length, perhaps more so than the particular type of cohesin, i.e. whether it is an SMC1α or SMC1β cohesin complex. Whether individual complexes prefer to associate with certain sequences or DNA structures along chromosomes is not known but not unlikely given the association of cohesin in mitotic cells with binding sites for transcriptional regulators. Synapsis is supported by all complexes, although to a different extent. It also remains unclear by which mechanism(s)–directly or indirectly–synapsis is promoted by cohesins beyond formation of an axis-loop-structure. Sex chromosome pairing at the short PAR is particularly vulnerable to loss of any cohesin, since all mutants fail in X/Y pairing. The previously observed dependence of X/Y pairing on cohesin dosage supports this notion [30]. Centromeric cohesion at the leptonema/early zygonema stage does not depend significantly on the meiosis-specific cohesins and thus relies on cohesion established during premeiotic S phase by SMC1α cohesin. Together with earlier publications it becomes clear that with progression of meiosis, cohesion increasingly depends on meiosis-specific cohesins, since SMC1α vanishes and SMC1β becomes prominent. Telomeres suffer from any absence of meiosis-specific cohesins, but the most from absence of RAD21L or REC8 with SMC1β. The mode of telomere protection, however, remains to be elucidated, but the TRF2 patterns revealed here by SIM hint at loop-like protective structures at spermatocyte telomeres. T-loops were initially described in 1999 [31], and TRF2-dependent t-loops recently demonstrated for somatic cells were indeed suggested to protect telomeres from non-homologous end-joining and ATM-triggered DNA damage signaling [28].

## Materials and Methods

### Mice

*Smc1β*^*–/–*^mice have been previously described [32, 33]. In *Smc1β*^*–/–*^mice, exon 10 was targeted representing 40% of the hinge domain. Generally, mice were bred and maintained in the animal facility of the Medical Faculty, Technische Universität Dresden (Dresden, Germany) according to institutional guidelines. All experiments were performed with approval by the State of Saxony. *Rad21L*^*-/-*^ and *Rec8*^*-/-*^ mice were generated as described previously [19, 34]. All mice were in the C57BL/6 genetic background. Number of mice used for the experiments: N = 5, Smc1β^-/-^; N = 4, Rec8^-/-^; N = 4, Rad21L^-/-^; N = 4, Smc1β^-/-^ Rec8^-/-^; N = 4, Smc1β^-/-^ Rad21L^-/-^.

### Single cell suspension and chromosome spreads

Surface-spread chromosomes were prepared by detergent spreading adapted from Wojtasz et al. [35]. Testis was taken from the sacrificed mice and tunica albuginea was removed. Tubules were digested in 1 ml of 1 μg/ml of collagenase type I—PBS buffer for 10’ at 32°C with slight agitation. Tubules were the centrifuged to pellet the cells and excess collagenase was removed. Pellet was then resuspended in 500μl of 0.025% trypsin and incubated for 5’ at 32°C. Then 200 μl of media with FCS was added to the Single cell suspension. Cells were then filtered through 40 μm to remove the cell debris and centrifuged. Pellet was then resuspended in 300 μl of PBS. Now single cell suspension was used for the chromosome spreads. 1.5 μl of single cell suspension were dropped on 7 μl of 0.25% of NP40. Cells were allowed to lyse for 2 mins and then fixed by adding 24 μl of S fix (1% paraformaldehyde, 10 mM sodium borate buffer pH 9.2). Samples were incubated for 1 hour at room temperature in a humid chamber. Slides were dried under a hood and washed two times for one minute with 0.4% Agepon (AgfaPhoto) and another three times for one minute with water. Slides were used immediately or kept at -20°C until IF staining.

### Testis cryosection

Testis were removed from sacrificed mice and placed in 2% (v/v) of formaldehyde/PBS for 40’ at RT for fixation before incubation in 30% sucrose/PBS overnight. Subsequently, testes were mounted in O.C.T (Sakura Finetek Europe), shock-frozen on dry ice and stored at -80°C. 8μm thick sections were made from the frozen testis, placed on the slides and dried for at least 30 min at RT. Then slides were treated with ice cold methanol for 10’ and 1’ with ice cold acetone. After completely drying, the slides were kept at –80°C or used immediately for the staining. The tubular stages were defined primarily based on cell associations and DAPI staining (centromeric and pericentric heterochromatin clustering) as described in [36].

### Immunofluroscence staining

Chromosome spreads and sections were treated in the same way. Slides were blocked with either blocking buffer (2% BSA, 0.1% Triton X in PBS) or 10% goat serum for at-least 1hr at RT before the primary antibody treatment. Slides were incubated with primary antibodies for at-least 3 hrs. at 37°C. Then slides were washed with blocking buffer and incubated with secondary antibodies for at-least 1hr. After the secondary antibody treatment slides were washed with blocking buffer and mounted with Vectashield containing 1μg/ml of DAPI. Statistics was performed using the 1-way Anova test, the Dunn’s test, the Whitney-Mann test or the Wilcoxon test as indicated.

### Immuno-Telo FISH staining

Telo-FISH of the G-strand was performed using the Telomere PNA FISH/Cy3 kit (Dako). The hybridization were done for 3 h at RT after denaturation at 80°C for 5 min. Cells from WT, SKO and DKO mice were always hybridized at the same time and compared with each other. Telomere intensity were obtained with equal exposure between all the genotypes and the relative length of telomeres was estimated by measuring the fluorescence intensity using ImageJ.

### Microscopy and image analysis

Fluorescence was visualized with Zeiss Axiophot fluorescence microscope and analysis of images was performed using ImageJ version 1.43u. Image analysis of SIM images was done using the 3D surface plot plugin in of ImageJ. Grid size and smoothing was kept as 256 and 10.0 values, respectively, for all images.

### Antibodies

The following antibodies (Tables 1 and 2) were used in this study:

**Table 2:** List of secondary antibodies used.

Secondary antibody	Conjugate	Source	Working concentrations (IF)
Goat Anti-mouse IgG	Cy3	Biolegends Inc.(405309)	1:500
Goat Anti-Rabbit IgG	Alexa Flour 488	Invitrogen(A11034)	1:500
Goat Anti-mouse IgG	Alexa Flour 488	Invitrogen(A11001)	1:500
Goat Anti-Mouse IgG	FITC	Cell signalling(101002)	1:500
Goat Anti- Guineapig	Alexa Flour 568	Invitrogen(A11075)	1:500
Goat Anti- Human	Alexa Flour 568	Invitrogen(A21090)	1:500
Goat Anti-Rabbit	HRP	Jackson Lab(111-035-003)	1:5000(IB)

## Supporting Information

S1 Fig (A) Staining for SYCP3 and gH2AX is shown for earlier (upper row) and the most advanced (lower row) stages for each genotype. The γH2AX forms one or two defined clouds in the most advanced stage. (B) Staining for SYCP3 and SYCP1 is shown for earlier (two left images) and the most advanced (two right images) stages for the two DKOs. SYCP1 indicates synapsis. (C) Staining for SYCP1 and (D) for HORMAD1 is shown for the extent of synapsis failure in the mutants (scale bar: 5 μm)
(TIFF)

S2 Fig Centromeres determined by immunofluorescence staining of spermatocyte chromosome spreads of WT, SKO and DKO mice. (A) samples were probed with anti-SYCP3 and anti-centromeric antibodies (ACA) (scale bar: 5 μm); red bars indicate SD. (B) Quantification of ACA signals. Statistically significant differences with a p-value >0.05 according to Dunn’s multiple comparison test are indicated.
(TIFF)

S3 Fig Cohesin localization, single channel images. Immunofluorescence staining of spermatocyte chromosome spreads of WT, SKO and DKO mice probed with anti-SYCP3, anti-SMC3 (scale bar: 5 μm).
(TIFF)

S4 Fig Cohesin localization, single channel images. Immunofluorescence staining of spermatocyte chromosome spreads of WT, SKO and DKO mice probed with anti-SYCP3, anti-SMC1β (scale bar: 5 μm).
(TIFF)

S5 Fig Cohesin localization, single channel images. Immunofluorescence staining of spermatocyte chromosome spreads of WT, SKO and DKO mice probed with anti-SYCP3, 4), anti-SMC1α (scale bar: 5 μm).
(TIFF)

S6 Fig Cohesin localization, single channel images. Immunofluorescence staining of spermatocyte chromosome spreads of WT, SKO and DKO mice probed with anti-SYCP3, anti-RAD21L (scale bar: 5 μm).
(TIFF)

S7 Fig Cohesin localization, single channel images. Immunofluorescence staining of spermatocyte chromosome spreads of WT, SKO and DKO mice probed with anti-SYCP3, anti-REC8 (scale bar: 5 μm).
(TIFF)

S8 Fig Cohesin localization, single channel images. Immunofluorescence staining of spermatocyte chromosome spreads of WT, SKO and DKO mice probed with anti-SYCP3, anti-RAD21 (scale bar: 5 μm).
(TIFF)

S9 Fig Cohesin localization, single channel images. Immunofluorescence staining of spermatocyte chromosome spreads of WT, SKO and DKO mice probed with anti-SYCP3, anti-STAG3 (scale bar: 5 μm).
(TIFF)

S10 Fig Frequency distribution of telomere length is shown for all the genotypes as measured using the ImageJ software. (TIFF)

S11 Fig SUN1 foci numbers for the indicated genotypes; red bars indicate SD. Those differences that are statistically significant with a p-value >0.05 according to the Dunn’s multiple comparison test are indicated.
(TIFF)

S12 Fig Super resolution (SIM) images of wild-type telomeres stained with anti TRF2, and of chromosome axes stained with anti SYCP3 as indicated. The sex chromosomes are marked by a blue arrow. Excerpts are provided showing examples of loop-like structures at the end of chromosomes.
(TIFF)

S13 Fig 3D surface plot analysis of wild-type and mutant telomeres of autosomes stained by anti TRF2 and anti SYCP3. High intensity signals are indicated by red color, low intensity by blue.
(TIFF)

S14 Fig Quantification of telomere features of wt and mutant spermatocytes. The percentages of chromosome ends showing telomeres in a loop-like pattern is provided, as is the percentage of chromosomes that show at one end 4, 3 or 2 telomere signals indicative of incomplete synapsis and/or failing cohesion. Further, the percentages of chromosomes that feature only one telomere signal, i.e. lack a signal at one end, and of chromosomes that display stretched telomeres, are given.
(TIFF)

S1 Table Summary of phenotypes observed in SKOs and DKOs. Synapsis is defined here as full synapsis between two homologs; the aberrant deposition of SYCP1 between sister chromatids or on a single chromatid is not considered synapsis. The degree of asynapsis in each mutant is indicated. The number of asterisks indicates the relative prominence of the phenotype. (1) Note: it is important to note that at later stages of meiosis, loss of cohesion is observed for meiosis-specific cohesin protein deficiencies.
(TIFF)
