# Supplementary figures and images for: Distinct Roles of Meiosis-Specific Cohesin Complexes in Mammalian Spermatogenesis
Source: PLoS Genet. 2016 Oct 28;12(10):e1006389. doi: 10.1371/journal.pgen.1006389 (PMC5085059; doi:10.1371/journal.pgen.1006389)

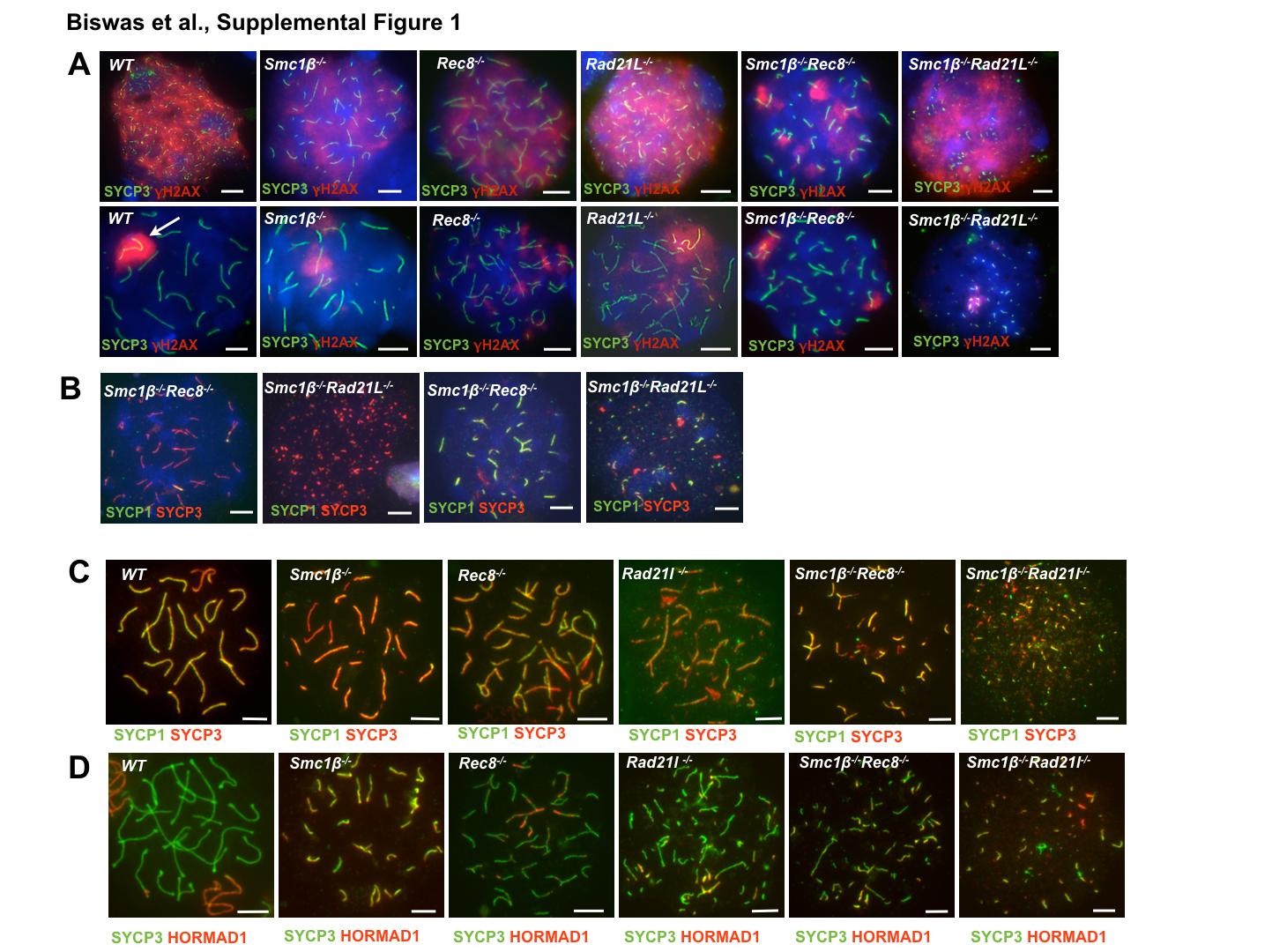

Supplement: S1 Fig — (A) Staining for SYCP3 and gH2AX is shown for earlier (upper row) and the most advanced (lower row) stages for each genotype. The γH2AX forms one or two defined clouds in the most advanced stage. (B) Staining for SYCP3 and SYCP1 is shown for earlier (two left images) and the most advanced (two right images) stages for the two DKOs. SYCP1 indicates synapsis. (C) Staining for SYCP1 and (D) for HORMAD1 is shown for the extent of synapsis failure in the mutants (scale bar: 5 μm) (TIFF) [file pgen.1006389.s001.tiff]

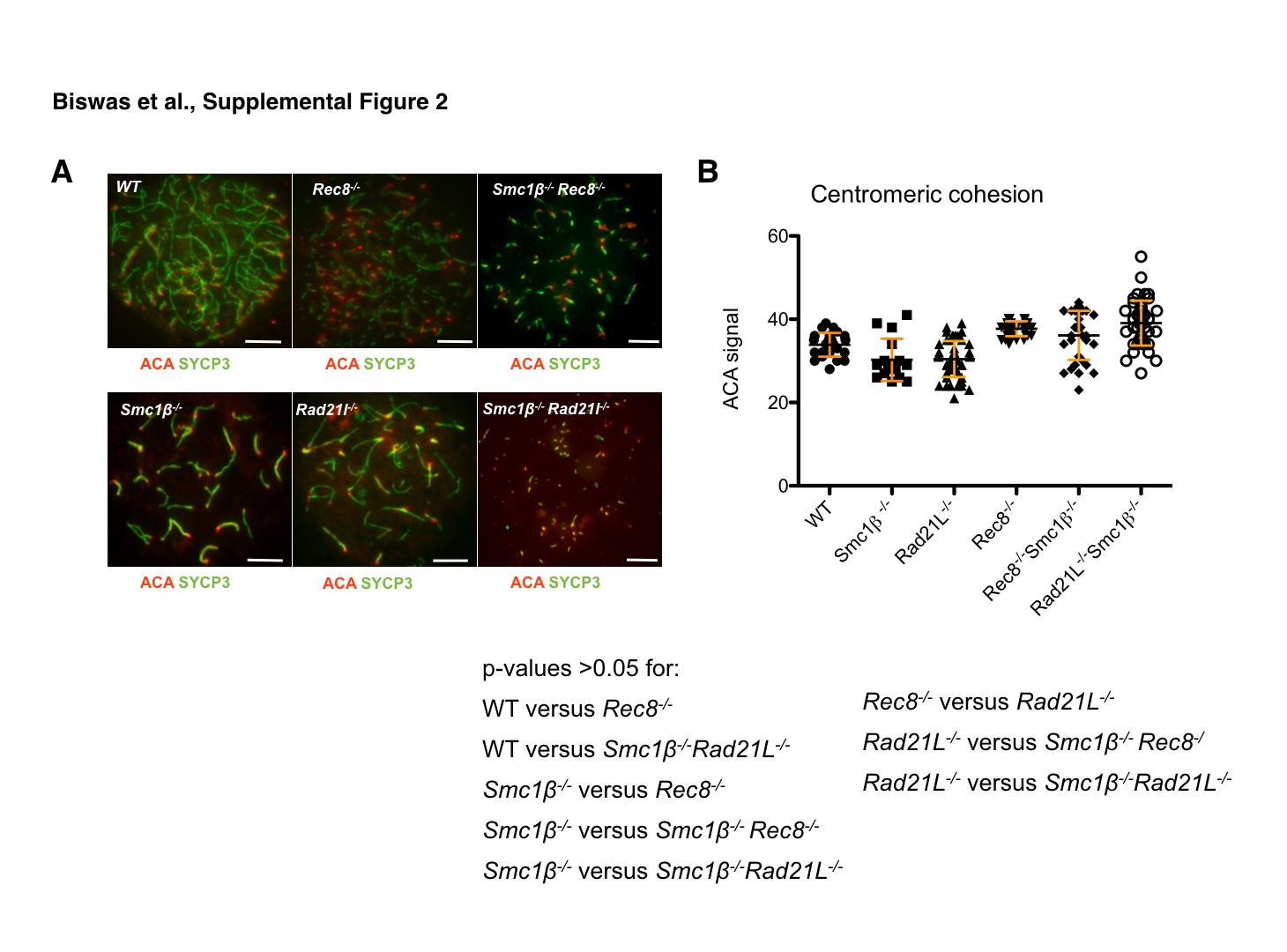

Supplement: S2 Fig — (A) samples were probed with anti-SYCP3 and anti-centromeric antibodies (ACA) (scale bar: 5 μm); red bars indicate SD. (B) Quantification of ACA signals. Statistically significant differences with a p-value >0.05 according to Dunn’s multiple comparison test are indicated. (TIFF) [file pgen.1006389.s002.tiff]

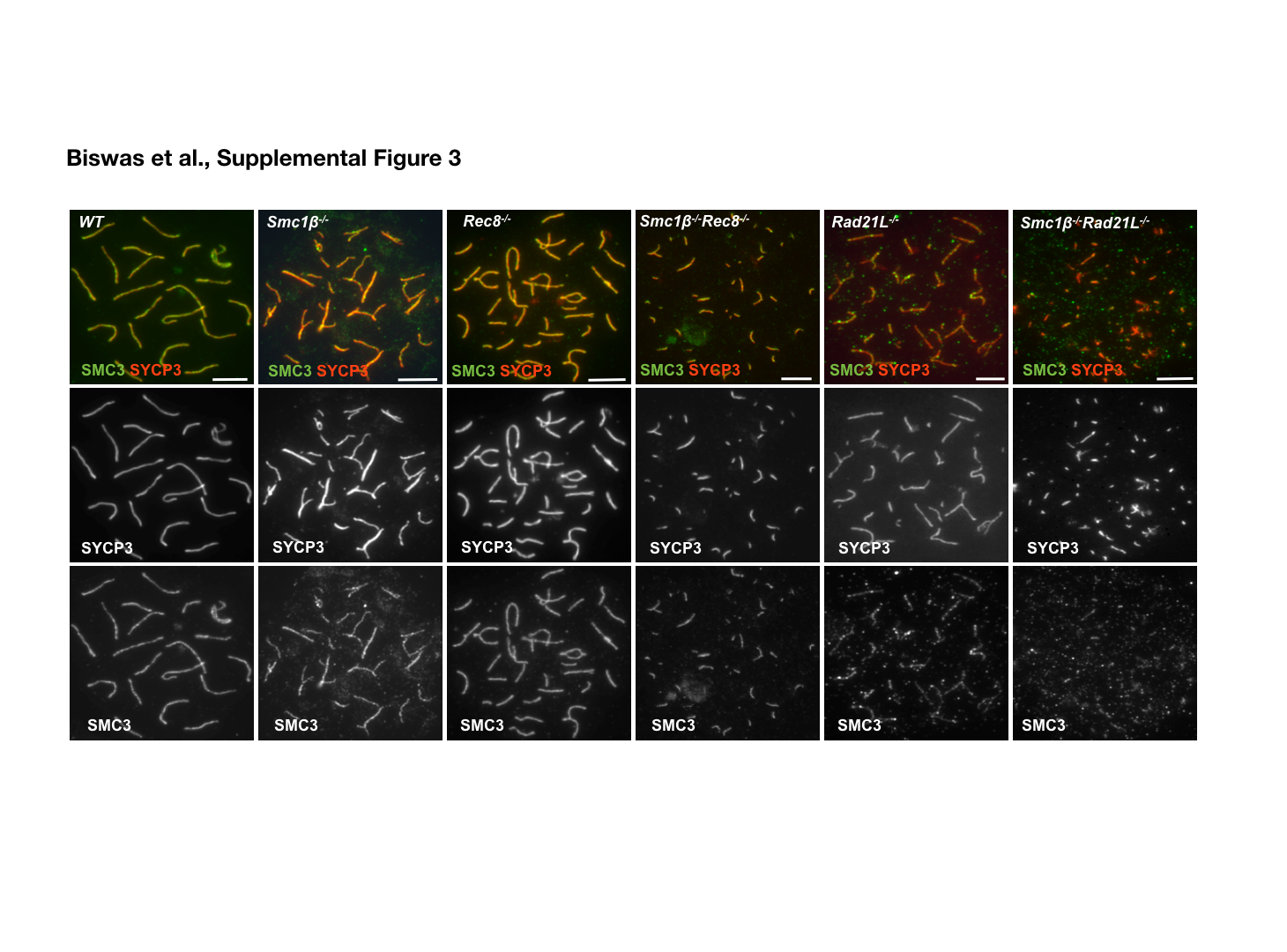

Supplement: S3 Fig — Immunofluorescence staining of spermatocyte chromosome spreads of WT, SKO and DKO mice probed with anti-SYCP3, anti-SMC3 (scale bar: 5 μm). (TIFF) [file pgen.1006389.s003.tiff]

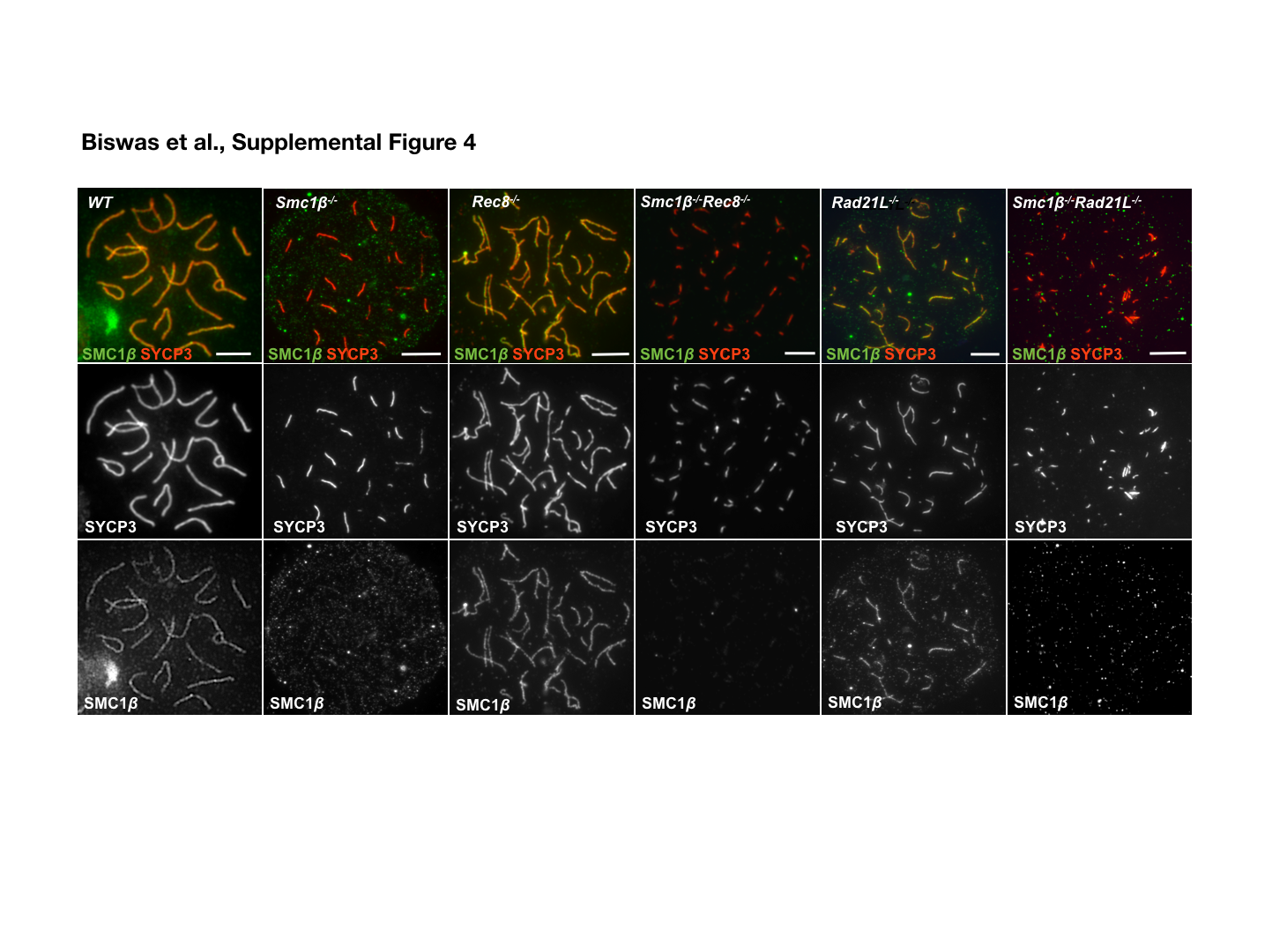

Supplement: S4 Fig — Immunofluorescence staining of spermatocyte chromosome spreads of WT, SKO and DKO mice probed with anti-SYCP3, anti-SMC1β (scale bar: 5 μm). (TIFF) [file pgen.1006389.s004.tiff]

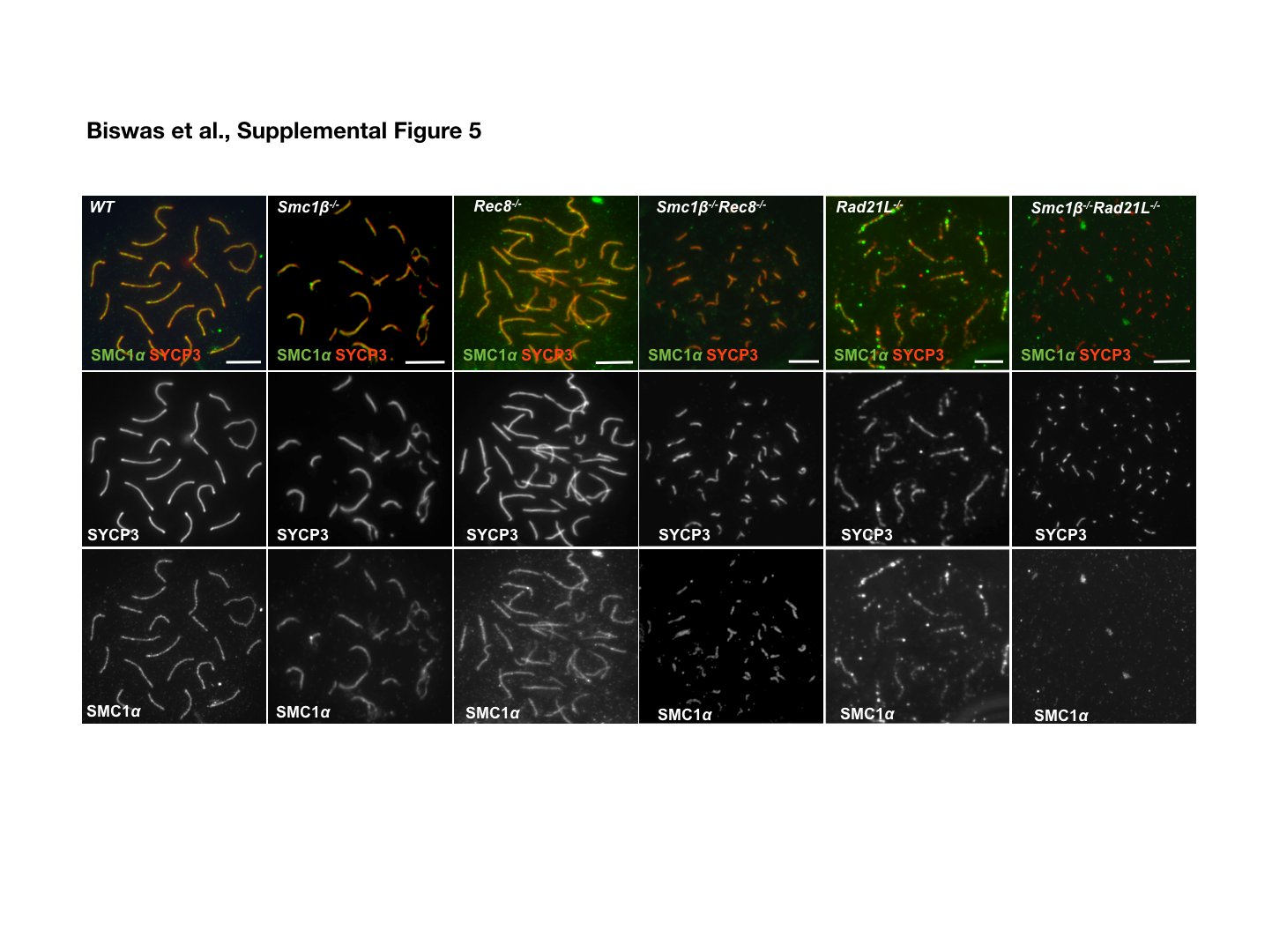

Supplement: S5 Fig — Immunofluorescence staining of spermatocyte chromosome spreads of WT, SKO and DKO mice probed with anti-SYCP3, 4), anti-SMC1α (scale bar: 5 μm). (TIFF) [file pgen.1006389.s005.tiff]

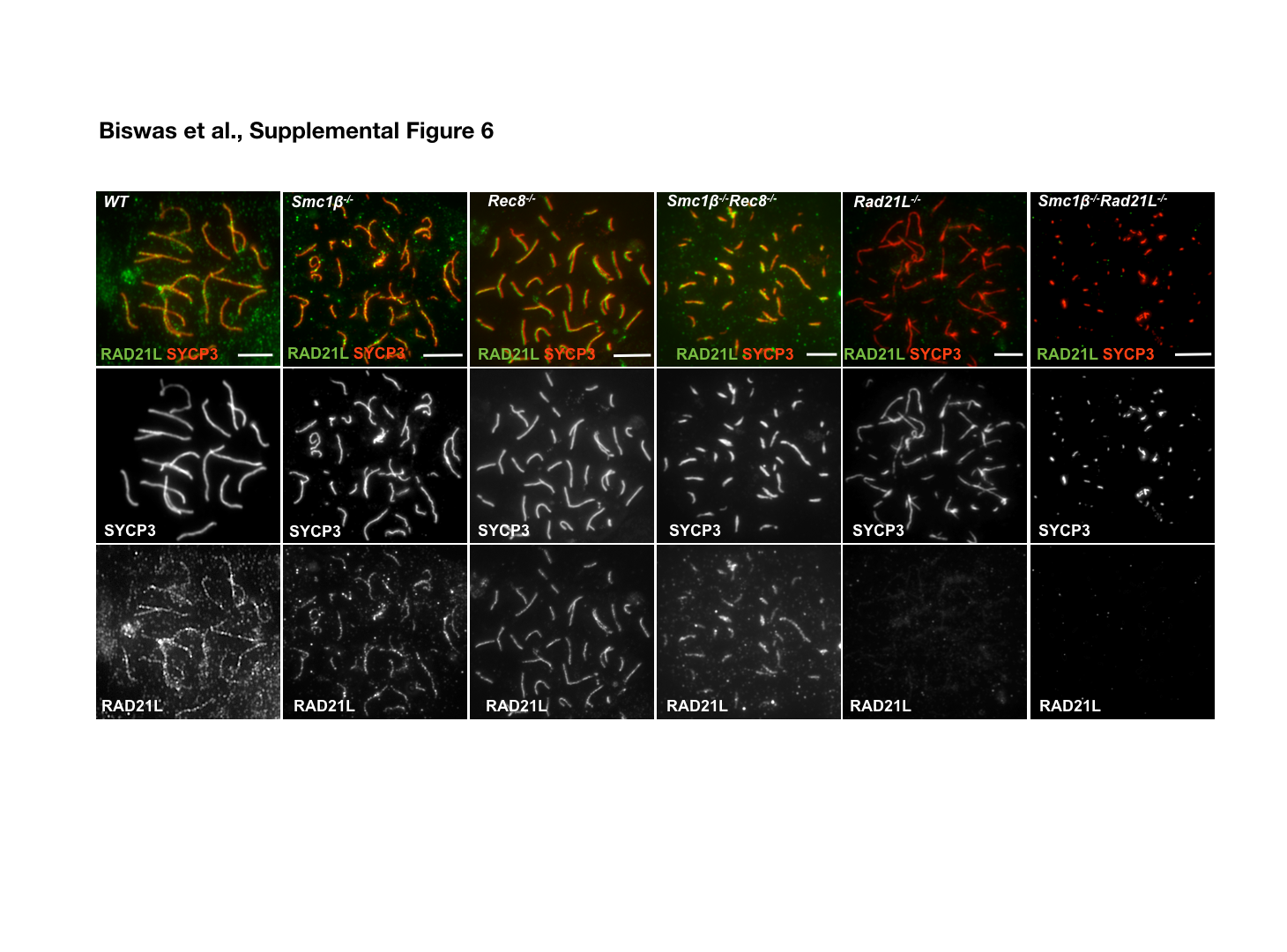

Supplement: S6 Fig — Immunofluorescence staining of spermatocyte chromosome spreads of WT, SKO and DKO mice probed with anti-SYCP3, anti-RAD21L (scale bar: 5 μm). (TIFF) [file pgen.1006389.s006.tiff]

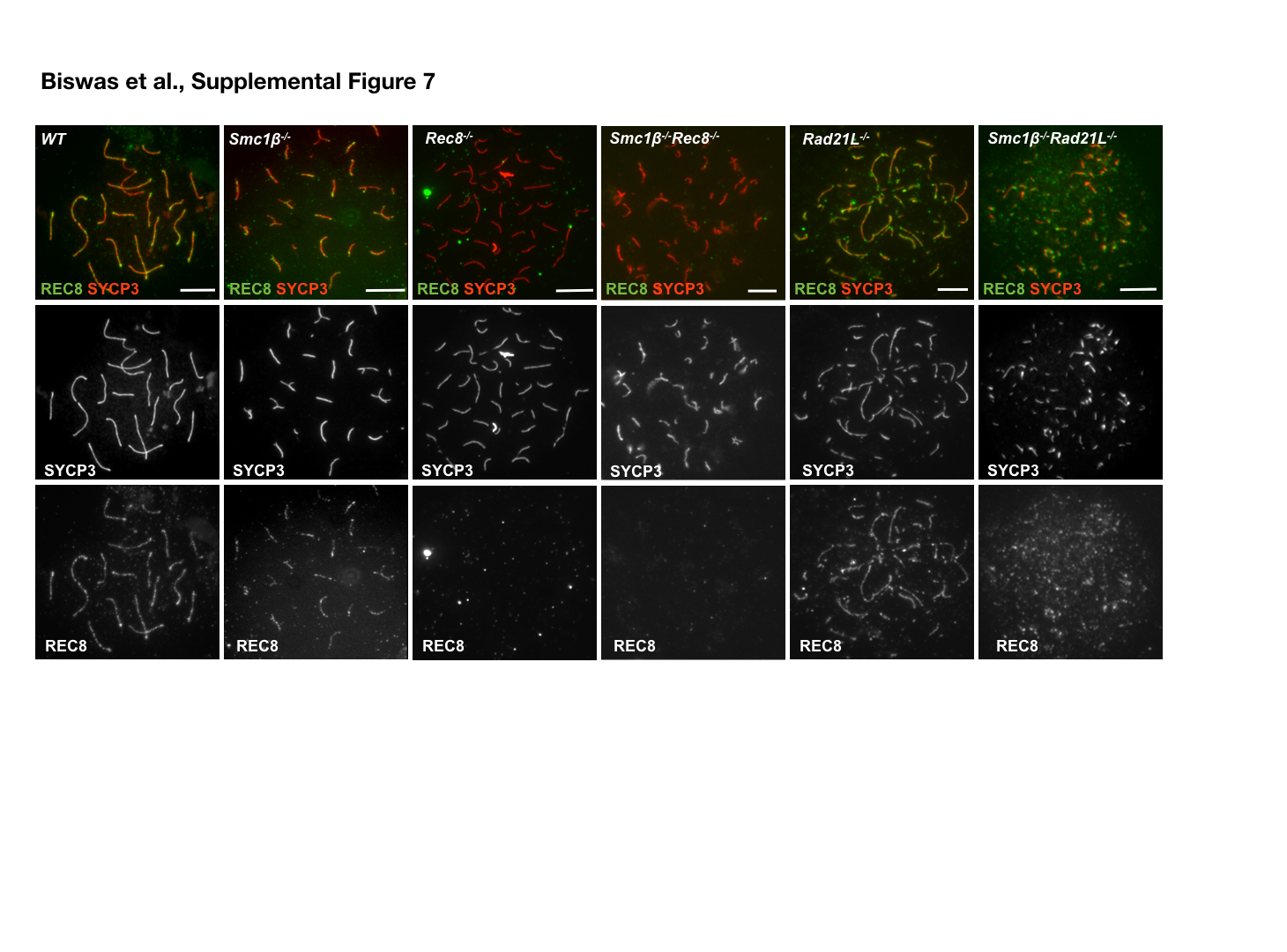

Supplement: S7 Fig — Immunofluorescence staining of spermatocyte chromosome spreads of WT, SKO and DKO mice probed with anti-SYCP3, anti-REC8 (scale bar: 5 μm). (TIFF) [file pgen.1006389.s007.tiff]

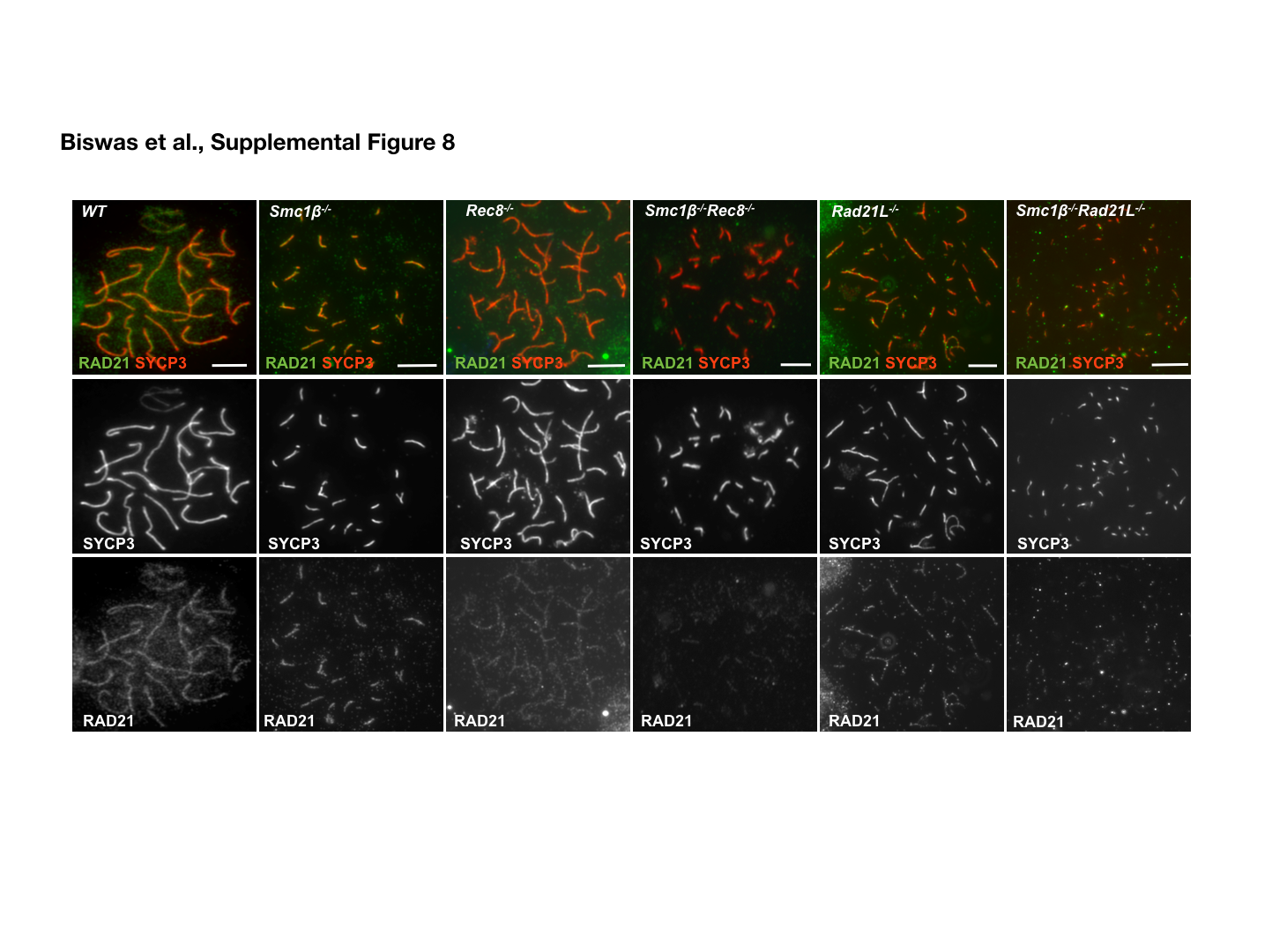

Supplement: S8 Fig — Immunofluorescence staining of spermatocyte chromosome spreads of WT, SKO and DKO mice probed with anti-SYCP3, anti-RAD21 (scale bar: 5 μm). (TIFF) [file pgen.1006389.s008.tiff]

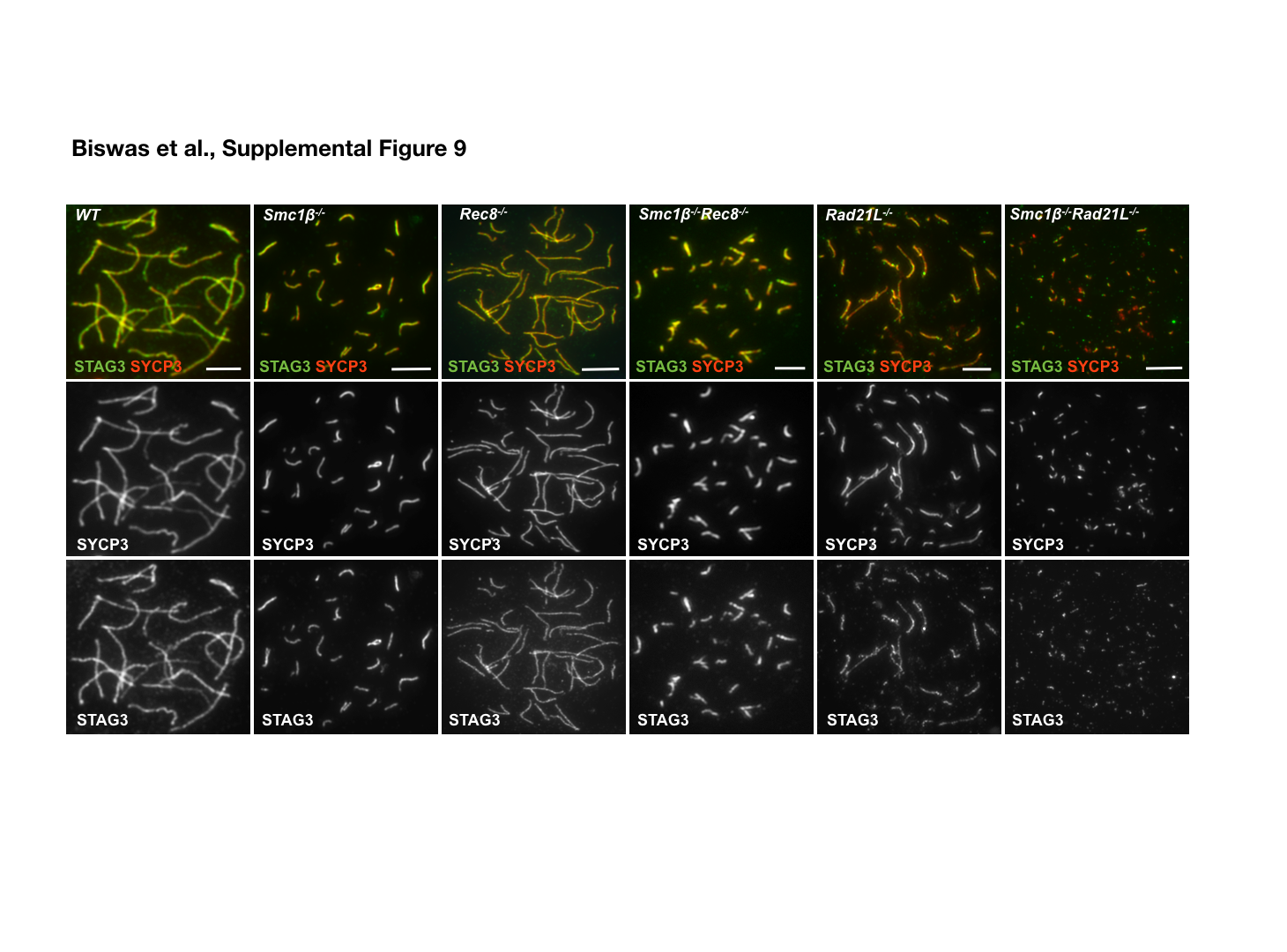

Supplement: S9 Fig — Immunofluorescence staining of spermatocyte chromosome spreads of WT, SKO and DKO mice probed with anti-SYCP3, anti-STAG3 (scale bar: 5 μm). (TIFF) [file pgen.1006389.s009.tiff]

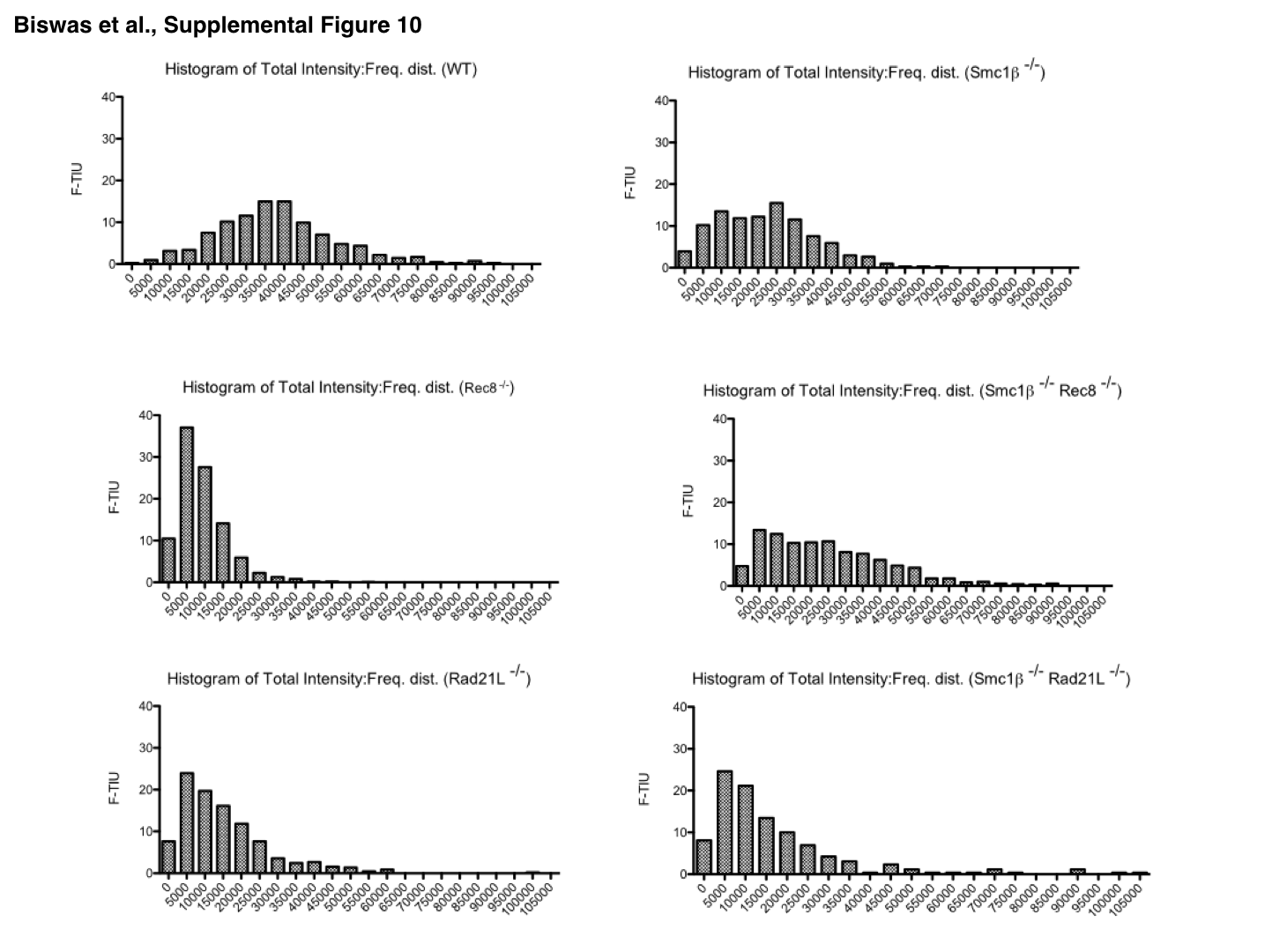

Supplement: S10 Fig — (TIFF) [file pgen.1006389.s010.tiff]

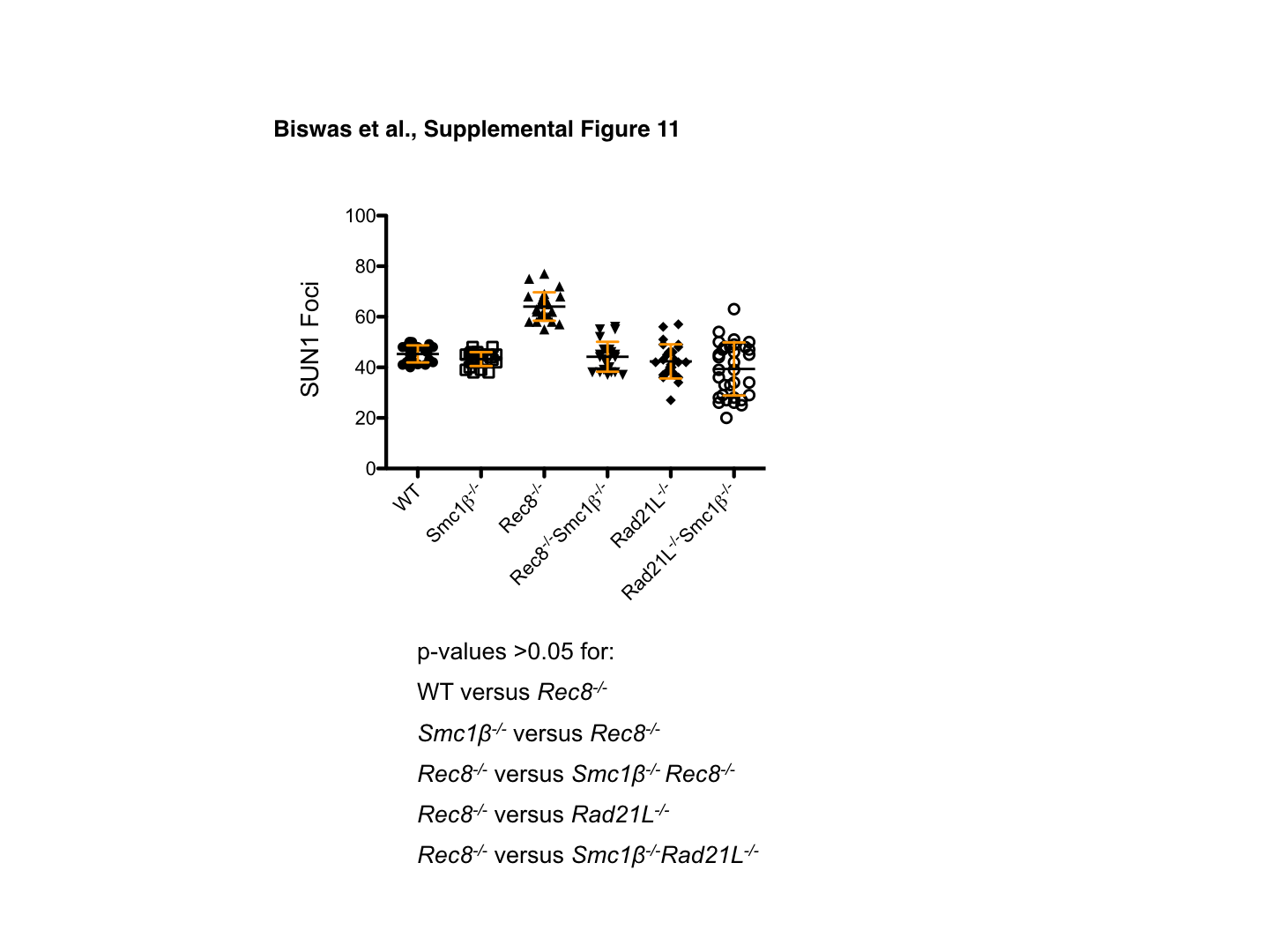

Supplement: S11 Fig — Those differences that are statistically significant with a p-value >0.05 according to the Dunn’s multiple comparison test are indicated. (TIFF) [file pgen.1006389.s011.tiff]

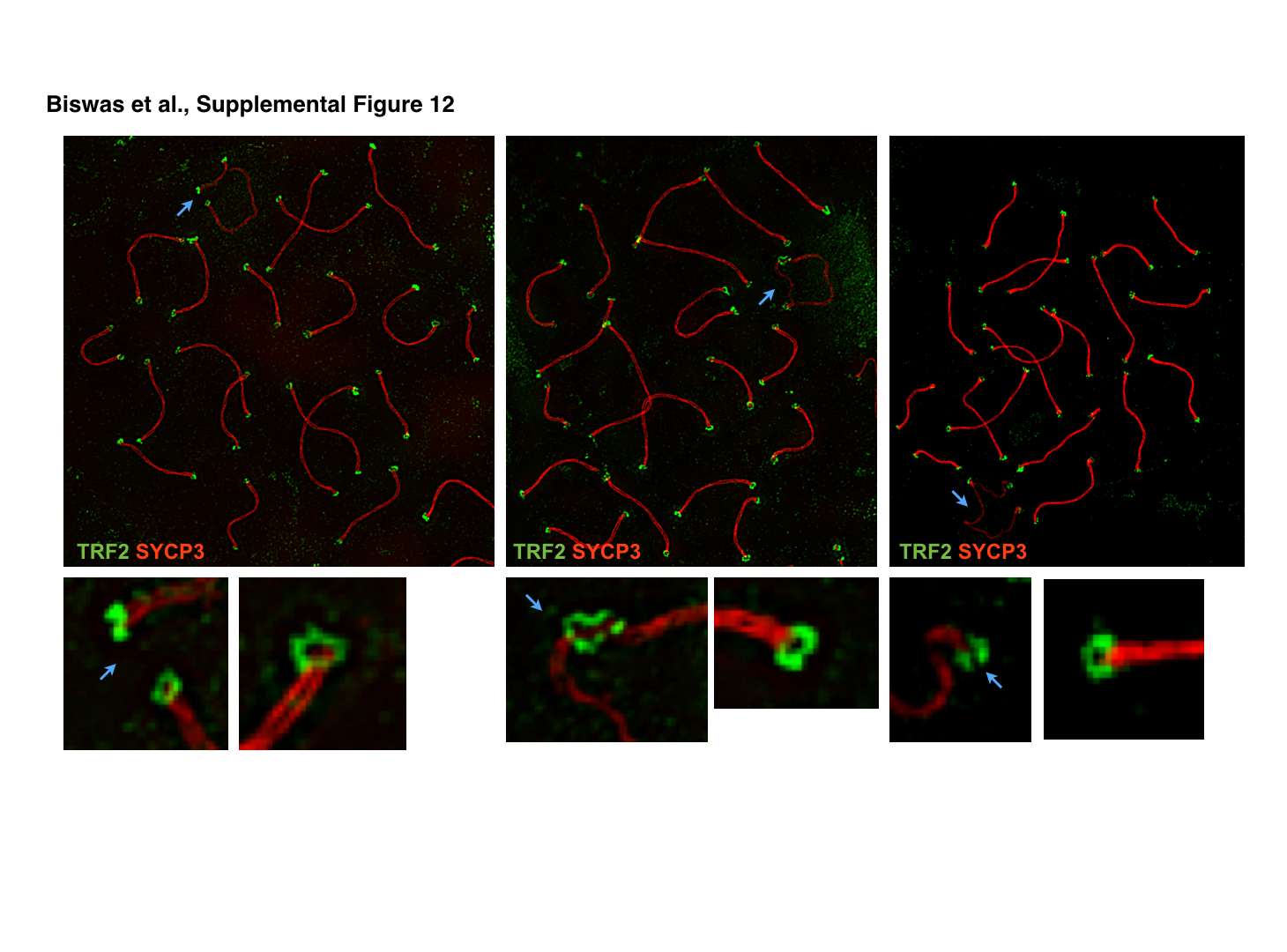

Supplement: S12 Fig — The sex chromosomes are marked by a blue arrow. Excerpts are provided showing examples of loop-like structures at the end of chromosomes. (TIFF) [file pgen.1006389.s012.tiff]

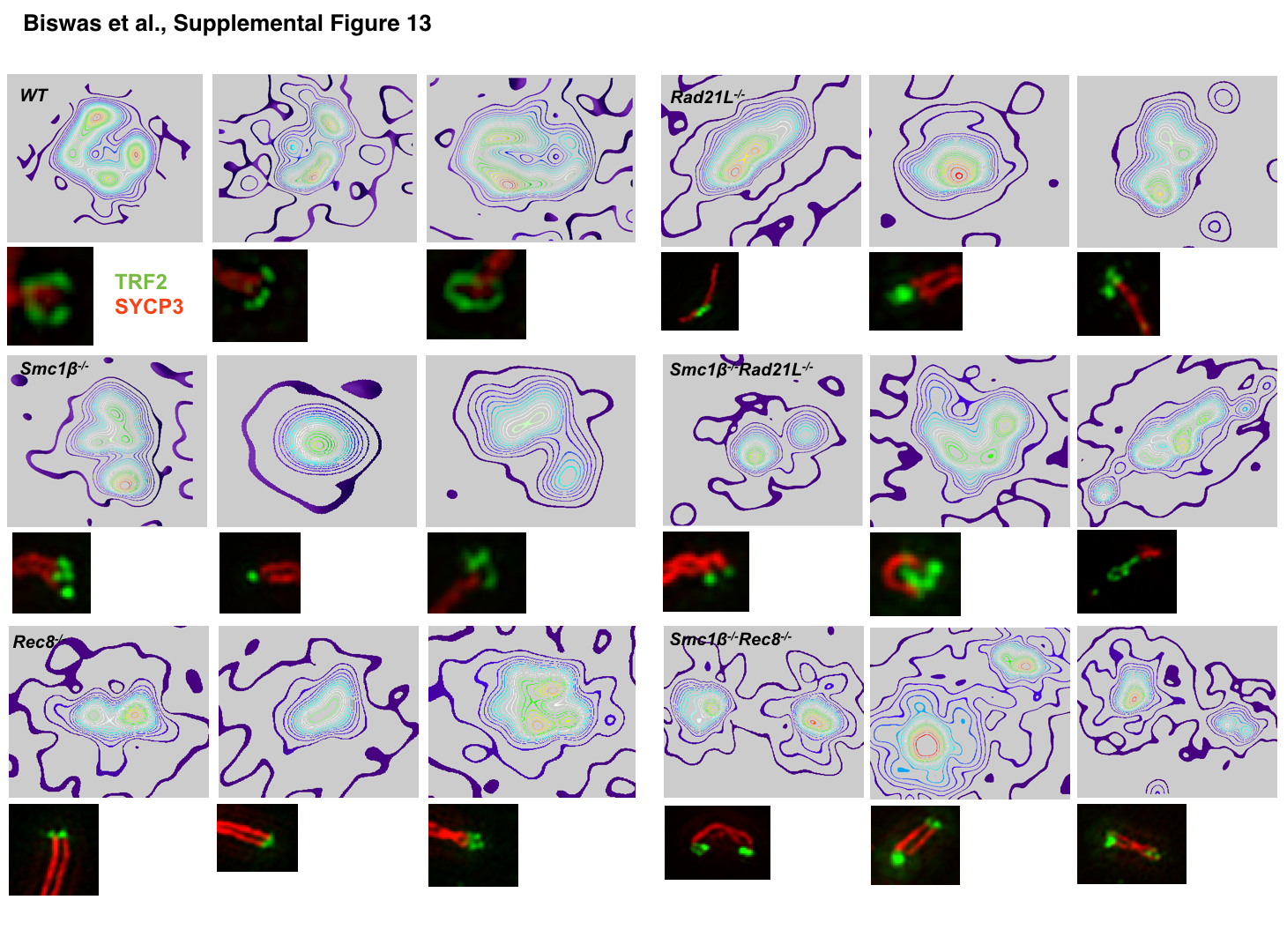

Supplement: S13 Fig — High intensity signals are indicated by red color, low intensity by blue. (TIFF) [file pgen.1006389.s013.tiff]

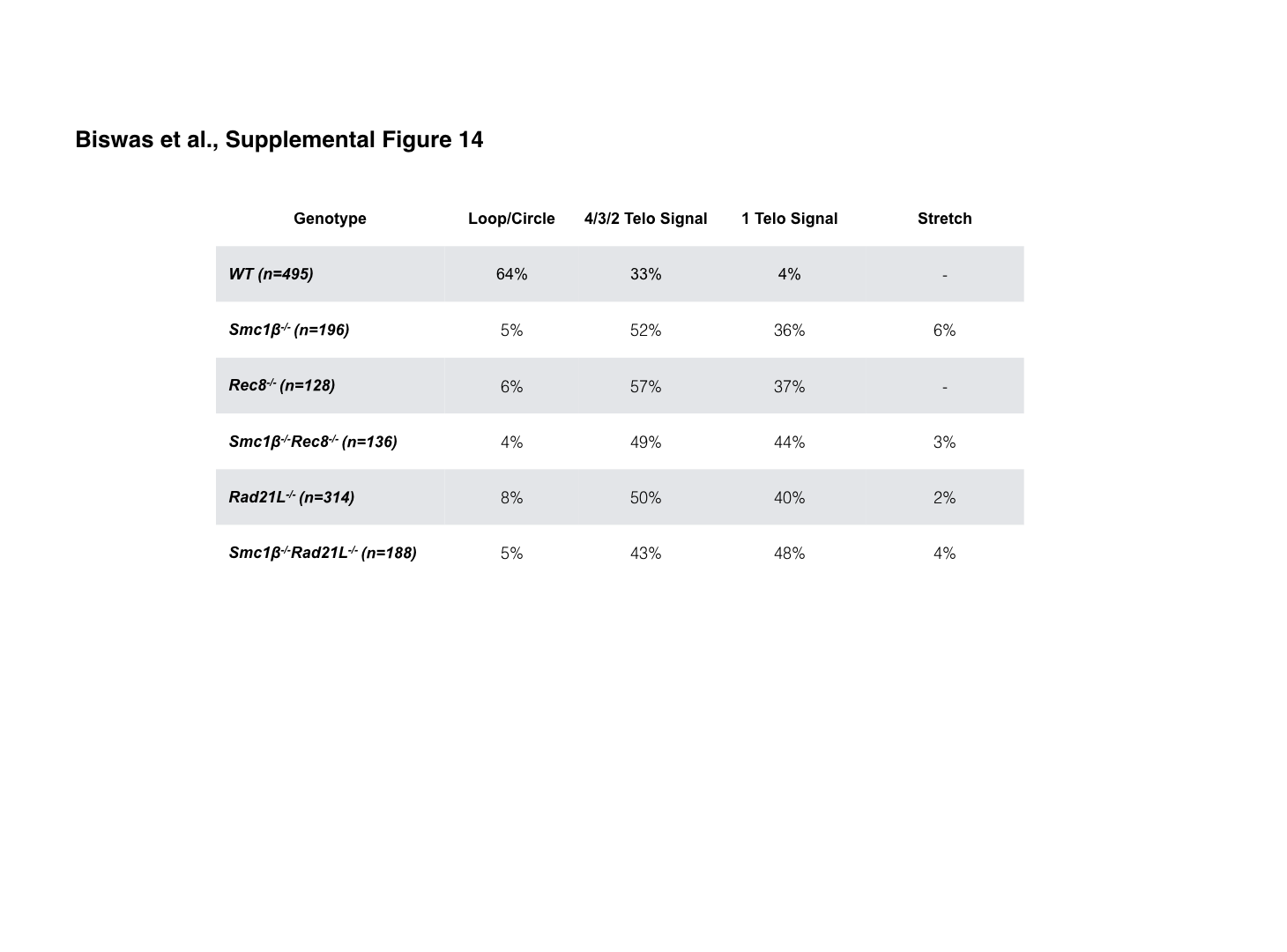

Supplement: S14 Fig — The percentages of chromosome ends showing telomeres in a loop-like pattern is provided, as is the percentage of chromosomes that show at one end 4, 3 or 2 telomere signals indicative of incomplete synapsis and/or failing cohesion. Further, the percentages of chromosomes that feature only one telomere signal, i.e. lack a signal at one end, and of chromosomes that display stretched telomeres, are given. (TIFF) [file pgen.1006389.s014.tiff]

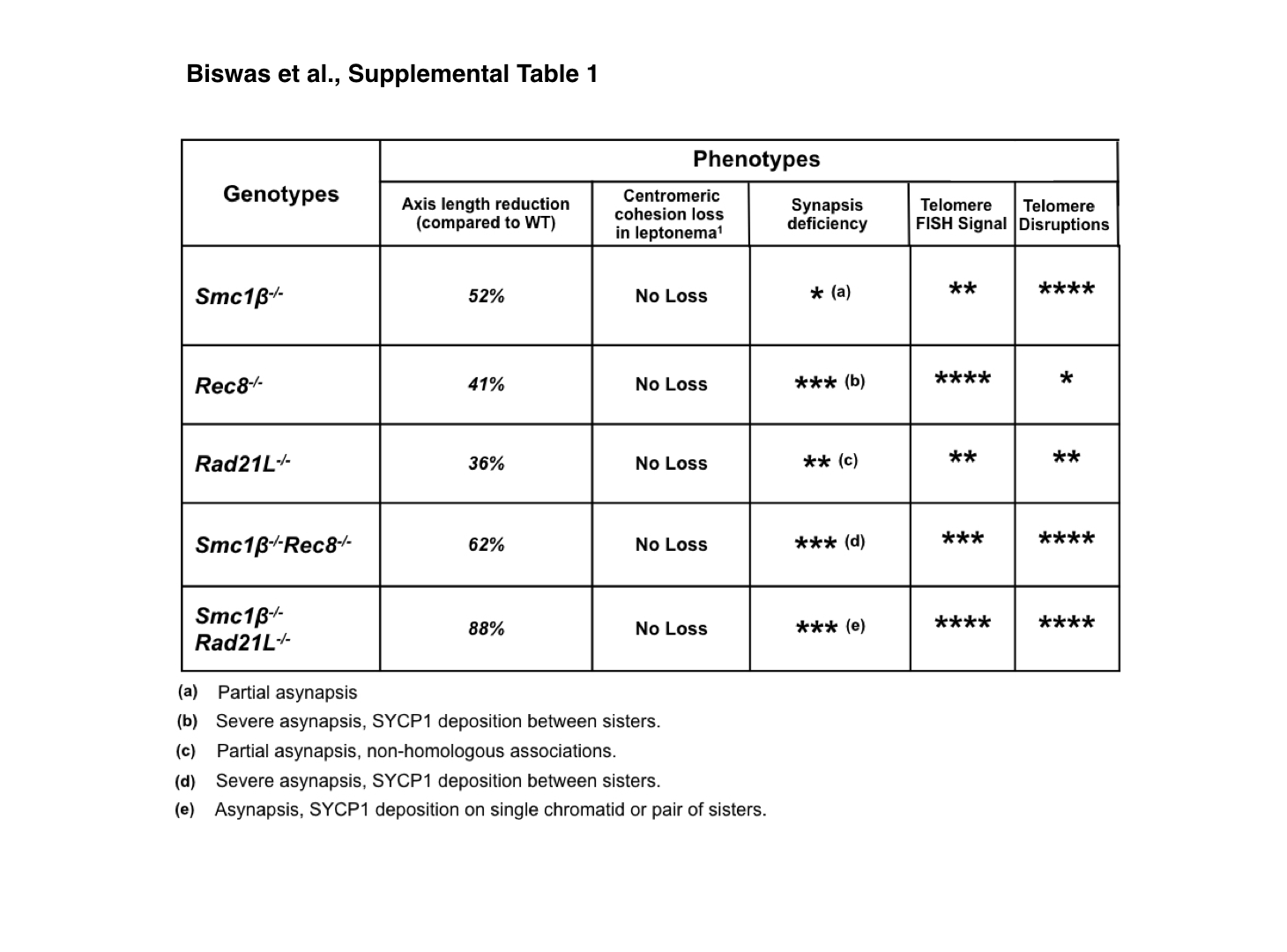

Supplement: S1 Table — Synapsis is defined here as full synapsis between two homologs; the aberrant deposition of SYCP1 between sister chromatids or on a single chromatid is not considered synapsis. The degree of asynapsis in each mutant is indicated. The number of asterisks indicates the relative prominence of the phenotype. (1) Note: it is important to note that at later stages of meiosis, loss of cohesion is observed for meiosis-specific cohesin protein deficiencies. (TIFF) [file pgen.1006389.s015.tiff]
